# A phenomenological study on the lived experience of men with Chronic Fatigue Syndrome

**DOI:** 10.1177/13591053231186385

**Published:** 2023-07-17

**Authors:** Gracie Elizabeth Snell, Catherine Heidi Seage, Jenny Mercer

**Affiliations:** Cardiff Metropolitan University, UK

**Keywords:** acceptance, chronic fatigue syndrome, diagnosis, health professionals, interpretative phenomenological analysis (IPA), lived experience, masculinity, norms

## Abstract

Whilst chronic fatigue syndrome (CFS) has been widely researched amongst women, studies investigating how men experience a CFS diagnosis is limited. This study utilised an interpretative phenomenological approach to interview five men who have a medical diagnosis of CFS. Six themes emerged to demonstrate the participants’ experiences prior to, during and after obtaining their CFS diagnosis. Findings revealed that participants were initially reluctant to accept their condition, confounded by their perception that symptoms compromised their sense of masculinity. They also felt that healthcare professionals had limited recognition of CFS leading them to seek social support and legitimisation from other sources. The struggle to come to terms with a different lifestyle and sense of masculinity prevailed. Such knowledge could be effectively utilised by researchers, practitioners and employers to facilitate an increased understanding of male accounts of the condition and more bespoke interventions where required.

## Introduction

Chronic fatigue syndrome (CFS), also referred to as *myalgic encephalomyelitis* (ME), is a multifaceted and poorly understood condition (The M.E Association, 2020). Prominent symptoms of CFS consist of lingering exhaustion, muscle aches, joint pain and cognitive dysfunction (Sandler and Lloyd, 2020). Additional CFS indicators include an light sensitivity, insomnia and swollen lymph nodes (National Health Service [NHS], 2020). Individuals with a mild form of CFS are generally able to carry out daily activities, although, as the severity of symptoms increase, this can be detrimental to daily functioning (Sandler and Lloyd, 2020). Prognosis for CFS is variable; some experience an improvement in symptoms after a couple of years, whilst for others, the condition is lifelong (Froehlich et al., 2022). The prevalence of CFS is estimated to be between 0.2% and 0.6% of the UK population (The M.E Association, 2020). CFS is more commonly diagnosed in females aged 40–60 (Sandler and Lloyd, 2020). The global CFS prevalence rate in females is approximately 1.31%, whereas for males, it is roughly 0.60%. To add, the National Institute for Clinical Evidence (NICE) corroborate the idea that CFS affects more females than males estimating a ratio of 4:1. The exact reason for this difference in prevalence is unknown (NICE, 2021).

Biological factors, such as differences in hormones or immunologic responses, may go some way to explaining sex differences in CFS prevalence rates (Lim et al., 2020). However, society-imposed norms surrounding the concept of masculinity could be an alternative explanation for these sex differences in prevalence, as these may influence whether males seek medical help (Loades et al., 2020). Males who adhere to the traditional norms of masculinity ideology, where ‘manliness’ is associated with being physically and mentally strong, commonly avoid accepting their ill health and can be influenced to hold dismissive and/or negative attitudes towards using healthcare services (Olanrewaju and Adeniyi, 2019).

CFS has also been historically characterised as a somatisation of psychological distress (Cheshire et al., 2021); a somatoform disorder are mental health conditions that cause an individual to experience physical symptoms in response to psychological distress (Abbasi et al., 2020). Classifying CFS as a somatoform condition may further inhibit the progression of males seeking medical advice as they wish to conform to a masculine ideal and not be viewed as unstable. When males do engage with healthcare services, there seems to be a contrast in symptom perception which is not commonly observed in women. Clarke (1999) contends that males attribute their symptoms to physiological exertion or heavy physical occupations, which possibly ties in with the traditional masculine norms discussed. Conversely, women are more likely to discuss stress as the perceived cause with their doctors. Hence, knowing that males are less likely to receive medical diagnoses for multiple reasons, it is plausible to suggest that in some cases CFS in males goes undetected. If so, prevalence rates are unrepresentative.

CFS is a challenging condition to diagnose and treat. The presentation of CFS overlaps with other chronic conditions and it is common for symptoms to be initially misdiagnosed as psychiatric in nature (Deale and Wessely, 2000). Emerging evidence suggests that the clinical manifestation of CFS may differ between genders, with males less likely to report chronic pain and have fewer immunological symptoms (Faro et al., 2016). Variances within symptom reporting are likely to reflect biological differences in pain perception (Fillingim and Maixner, 1995) and the gendered nature of symptom appraisal and assessment (Van Wijk and Kolk, 1997).

The nature of CFS can also lead to *delegitimation* (Ware, 1999). For instance, fatigue is commonly perceived to be a consequence of a busy lifestyle which in turn, creates the illusion that a person with CFS is not seriously ill as they attempt to continue their daily functions. Additionally, there remains a perception that the symptoms of CFS are ‘made-up’ or exaggerated to escape the responsibility of duties that are perceived undesirable by society, leading individuals to be questioned regarding their level of competency (Ware, 1999). Dickson et al. (2007) suggests that delegitimising experiences within CFS may be dually sourced: the construction of the symptoms by other people (colleagues and partners for example), and the perceptions of health professionals (HPs) regarding the underlying cause. Their phenomenological study with eight females and six males found this to be a factor amongst participants from both sexes.

Wilde et al. (2019) offer one of the few experiential studies which focus exclusively on males experiencing CFS. Using phenomenology and a photo elicitation task, 10 males who had been diagnosed with CFS were asked to upload photographs representing their perception of being male and living with CFS. They were then interviewed about their experiences and asked to comment on their photographs. Findings were structured around three interrelating themes which explored ‘the loss of a masculine identity’, ‘the marginalisation attached to CFS and masculinity’ and ‘coping with and adjusting to a dual identity’ (as being male and having a CFS) through assimilation and acceptance. The study provides an insight into some of the unique challenges which men face, such as difficulty in receiving support both socially and from HPs. The authors attribute this to the stigma experienced when attempting to contend with both personal and societal expectations to self-manage an illness. It also illustrates how through improved self and peer understanding of the illness, participants were able to adapt their perception to threats which might have previously jeopardised their masculinity.

Based on the literature reviewed above, it is evident that there is limited understanding of how males experience CFS. It is important to look exclusively at them as a group to gain an understanding of their experiences and expand the knowledge base regarding males with CFS. It is only then that meaningful comparisons can be made with research focusing on females to establish if sex differences do (or do not) exist. Therefore, the aim of this study is to gain a phenomenological understanding of the male experience of living with CFS. The focus is on seeking medical advice, accepting a diagnosis and receiving support. All of which have typically been highlighted in the literature reviewed as areas with which males struggle when experiencing a chronic illness.

## Methodology

### Design

A qualitative approach, using semi-structured online interviews and an interpretative phenomenological analytic (IPA) methodology, was adopted to explore the experiences of males living with CFS. Online interviews were selected due to the geographical dispersion of participants, as well as the COVID-19 lockdown restrictions deterring in-person interviews at the time of data collection. A semi-structured approach to interviewing allows more flexibility than a structured interview, which is important when undertaking experiential research; this in-depth approach has become the default for IPA studies (Smith and Nizza, 2022). Additionally, semi-structured interviews are recommended in cases of vulnerable participants, such as those with a chronic health condition in the current study (Husband, 2020).

### Participants

An opportunistic, purposive sampling method was used by selecting participants on a first come, first served basis. In keeping with guidance relating to IPA, a small homogenous group of five participants were interviewed. This gave detailed and manageable accounts which represented the experiences of each participant. Such a sample size further illustrates the idiographic commitment of IPA which is highly useful for analytical purposes (Smith and Nizza, 2022; Smith et al., 2009). The seven individuals not selected were then allocated as reserves, ready to take the place of any withdrawn participants. The average participant age was 42.

The inclusion criteria specified that all individuals must be cisgender male and aged 18 plus. Participants must have been clinically diagnosed with CFS within the past 10 years, as events recalled from more than 10 years ago have a higher chance of being distorted (Deck and Paterson, 2021). Also, as CFS guidelines have remained relatively consistent throughout the past 10 years, interviewees will be reflecting on the same diagnostic procedure (Sandler and Lloyd, 2020).

To protect confidentiality and anonymity (BPS, 2017), participants were allocated identification numbers and referred to by pseudonyms within the studies’ write-up. Participant identification and interview records were stored on a password-protected device. Key demographic details are captured in Table 1.

**Table 1.** Key demographic characteristics of participants.

Participant	Age	Sexuality/relationship status	Ethnicity	Occupation
Tyler	55	Gay, Long-term Partner	White British	Business Owner
Dave	32	Heterosexual, Married	White British	Commercial Lawyer
Sam	32	Heterosexual, Long-term Partner	White British/New Zealand Origin	Registered Social Worker/Part-time PhD Student
Paul	42	Heterosexual, Married	White British	Ex-Teaching Assistant
Simon	45	Heterosexual, Married	White British	Not in employment/education

### Procedure

Recruitment took place over a 4-week period by publishing an online advert on the Facebook page ‘CFS/M.E.- Increase Awareness & Understanding’. Permission was gained from the administrator prior to posting the advert. Potential participants were provided with a consent form and information sheet which described the aims of the study, details surrounding the withdrawal process and how any potential ethical issues were managed. All participants gave written consent to take part in the study.

The interview schedule was developed based on an exploration of existing relevant literature highlighting the impact of CFS, and with open-ended questions to collect participants’ experiences (Smith et al., 2009). It began with the least-sensitive questions, such as ‘how did you become diagnosed with CFS?’, to build trust and rapport. After establishing a basic understanding of the participants’ CFS history, the schedule used a funnelling method to focus on more specific issues by using open-ended questions and prompts to elicit rich participant-led discussions (Kiger and Varpio, 2020). For example, ‘How do your family, friends and employers respond to your condition now?’. The questions posed to participants provided the opportunity for descriptive accounts of their diagnostic experiences, with reference to the role of medical professionals and healthcare services. Despite the use of prompts, an *inductive approach* was adopted (meaning that the interview schedule was not necessarily followed in any strict or rigid way).

Individual interviews were carried out and recorded over a 2-week period using the platform *Zoom*. Following the interview, participants were emailed a debrief form with contact details for mental health and CFS support services, as well as a reminder of the opportunity to withdraw.

### Data analysis

Interview recordings were transcribed using *Otter.ai*, a software that converts recorded speech to text transcription. IPA was used to analyse the data and examine the lived experiences in a hermeneutic style (Boden et al., 2019). IPA was selected rather than thematic analysis as the nature of IPA is highly effective when there is limited research in the topic area, such as CFS in males (Braun and Clarke, 2021).

The framework provided by Smith et al. (2009) was followed. Interviews were read thoroughly by GS (the first author) before completing the initial coding process based on a preliminary interpretation of the experiences discussed. A search for patterns amongst these initial ideas were identified and recorded as *emergent* themes (Smith et al., 2009). Whilst considering convergence and divergence, further connections were made among these emergent themes which led to the establishment formulated themes (Smith et al., 2009). GS led on the analytic process eliciting regular discussions with co-authors around the appropriateness of the emerging theme names, quotations and nature of interpretative commentaries presented. This aided reflection and the reviewing of themes.

## Results

The following six themes illustrate participants’ journeys from acquiring a formal CFS diagnosis, the emotions felt when receiving the diagnosis and their experiences of living with the condition. Findings are documented through verbatim extracts from transcripts supported by authors’ interpretative commentary with links to extant research.

### Fighting the symptoms to maintain male competency

Participants were initially reluctant to seek medical help for their symptoms, supporting the findings that men commonly avoid disclosing their health struggles (Richardson et al., 2022; Ross et al., 2020). Given this reluctance, participants demonstrated various coping strategies, such as continuing with routine sporting activities, pursuing University commitments and consuming energy drinks to combat the fatigue. When Tyler and Sam described their initial approach to dealing with CFS, both used distinct metaphors associated with masculinity:

Before going to the doctors, I remember trying to just battle through it [Tyler, L32-33]

And

. . ..I just tried to muscle my way through my hectic lifestyle [Sam, L14-15]

The metaphors ‘battle’ and ‘muscle’ reflect norms around strength and courage being important aspects of male dominance within Western society (Connell and Messerschmidt, 2005). The use of ‘battle’ also has connotations of men fighting at war; Tyler was going to fight the illness, rather than admit to being unwell.

These males seem to be exhibiting behaviours which conform to the stereotypes of hegemonic masculinity. This led to a reluctance to accept being ill, something which is also shown in Paul’s disclosure:

I didn’t want a diagnosis at first you see. I simply wanted to carry on playing football with the lads and going to the pub. I couldn’t say no because that would be embarrassing and they (his friends) would just take the mick and tell me to ‘man up’ [Paul, L4-7]

Similarly, concern about what others might think was evident in Simon’s statement:

I didn’t want to be seen as ‘weak’ by them [Simon, L49]

Such accounts suggest that men with CFS attempt to conceal their illness to avoid reactions which could be stigmatising and marginalising. This resonates with Wilde et al.’s (2019) findings that males with CFS feel that the condition negatively impacts on their capability to maintain a desired level of masculinity.

### Perceptions of healthcare inequality

Participants seemed to have preconceptions surrounding the level of support that males receive. This also confounded their desire to seek medical help and likely contributed towards prolonged periods before doing so. Sam disclosed his belief that males do not receive adequate healthcare, which led him to be wary of visiting the GP:

There has been so much publicity on how males are not taken seriously when they admit how they are feeling, so this puts me off going [Sam, L192-194]

Paul felt that males are treated with less respect than females; this was attributed to an assumption that females require healthcare support for an illness, whereas men are ‘strong’ enough to overcome a health struggle without professional help:

I feel the health services are not male friendly. I feel that males are meant to be the ‘strong alpha male’ and so we are perhaps treated less sympathetically than females [Paul, L33-35]

Paul’s account reflects a belief that inequality exists in the way men with chronic illnesses are considered when they seek advice from a HP. It also re-iterates the social norm of males being both physically and mentally strong (Connell and Messerschmidt, 2005).

### Limited professional recognition of CFS symptoms

All participants experienced frustration about the length of time it took for their on-going physical suffering to be recognised by HPs and treated as CFS. They frequently reported that medical professionals assumed their health concerns were due to existing psychological infirmity, such as long-term depression. One participants’ GP informed him that he was simply manifesting the physical pain because of his poor mental health. Adding to this, two participants were misdiagnosed with clinical depression and recommended anti-depressants to alleviate their symptoms; a common encounter that females experience during initial GP consultations for CFS (Geraghty, 2017). Simon’s GP attributed symptoms to chronic migraine and Dave was referred to both insomnia and irritable bowel syndrome (IBS) specialists. Such examples support the idea that CFS is commonly misdiagnosed (M.E Research, 2021). Despite this, participants discussed a sense of being ‘in tune’ with their own bodies, which meant they were adamant that the symptoms were not a result of any physical or psychological condition other than CFS. Sam felt that the GP labelled his symptoms as CFS but despite 10 years of research into the condition, was not fully accepting of it:

My GP, kind of accepted and treated me as if I had chronic fatigue, but I still think he (GP) probably, even 10 years later, probably thinks that it is mainly an element of psychological stuff going on, which is just simply not the case [Sam, L35-38]

This extract by Sam also indicated that he still believes that HPs have a general lack of CFS awareness and that his illness is not psychological in nature. Participants often felt that HPs did not wish to accept CFS as a legitimate illness; they reported feeling that GPs thought they were imagining or exaggerating the symptoms due to being psychologically unstable. This has also been reported by females with CFS (Karfakis, 2018). Tyler reported that his GP even termed CFS as ‘pseudo M.E’. which profoundly implies that the illness is not genuine:

He (GP) called it ‘pseudo M.E’ and I was like no, this is not in my mind. I am not making it up or imagining the pain [Tyler, L29-31]

It is evident that the lack of CFS recognition from HPs caused a delay in receiving a formal diagnosis. As a result, participants commonly reacted with feelings of anger and hatred towards HPs with regards to these problematic clinical encounters. Consequently, the mutual respect and communication associated with achieving a good doctor-patient relationship was often eroded.

Simon’s exasperation with GPs is revealed in the following quotation:

I could hardly stand up or even raise my arm in the air. Even at this point, I was STILL going to the doctors and they (GPs) STILL said ‘well you have got here ok, so you must be absolutely fine. There’s nothing I can do’ [Simon, L52-55]

The repetition and emphasis of the word ‘still’ by Simon demonstrates the length of time it took for his illness to be acknowledged. The lack of faith in the GP is also demonstrated by Simon:

One doctor point blank said to me that ‘CFS doesn’t exist’ when I suggested to him that this (CFS) could be what I am suffering from. Crazy [laughs] I even changed my GP surgery after that as I had just had enough [Simon, L73-77]

Simon’s annoyance with his GP is clear in the above extract, so much so that he felt the need to change surgeries in the hope of receiving satisfactory support. Other participants, like Simon, perceived such diagnostic challenges as personal attacks on their sense of morality. For instance, some felt accused of searching for an escape from their day-to-day responsibilities, such as ‘leaving work early’ (Dave, L30) or ‘not working at all’ (Simon, L45), which further implies that the condition has connotation with being ‘lazy’.

It should be acknowledged that a CFS diagnosis is only given when symptoms have been continuously present for at least 3 months (NICE, 2021), indicating that receiving a diagnosis is generally a lengthy process. As illustrated earlier, there was also a reluctance amongst the participants to seek medical intervention in the early stages of the illness, which would also prolong the diagnostic process. However, for some participants, the time to diagnosis significantly exceeded the recommendations by NICE (2021). For instance, it took Tyler roughly 8 years and Glen 4 years to be formally diagnosed.

### Challenges in accepting a new life

Having acceptance, validation and acknowledgement from HPs was highly significant. Diagnosis of CFS offered reassurance and legitimised the symptoms experienced. Indeed, a diagnosis prompted the need to move forward with their lives:

. . ..having a diagnosis gave me an answer and closure [Paul, L10]

And

I almost felt a sense of relief knowing that I wasn’t making it up (the symptoms) and it was real. I could finally accept the new me [Simon, L68-70]

However, with acceptance also came an acknowledgement that the participants’ lives had changed. Paul felt a sense of guilt, and disclosed a concern about the impact of CFS on his marital relationship:

I almost feel guilty, like I’ve stolen something from her by me being ill. I worry then that she will stop loving me [Paul, L51-53]

Support for the work of Wilde et al. (2019) that the burden which CFS creates often goes unrecognised, can be found in Tyler’s statement:

Sometimes I feel invisible, and my old life has been snatched from me like some kind of punishment [Tyler, L119-120]

Here, Tyler’s reference to being punished by CFS is played out through it having ‘snatched’ his life. His comments mirror Paul’s use of the term ‘stolen’ to explain how CFS has taken something away from his life.

The sense of loss that is expressed here, was also evident when participants talked about a loss of masculinity.

### Challenges to masculinity

Participants reported that living with CFS symptoms impacted negatively on their ability to meet expectations of masculinity even when they had a diagnosis; a strong sense of dissonance between the reality of their lives and the perceived norms they sought to live up to remained:

I felt I need to be that male breadwinner, you know, I want to provide, want to kind of be a successful father for my kid [Sam, L183-185]

Income here is a further marker of masculinity and consequently, one’s level of control over their financial stability (Herron et al., 2020). Both Sam and Paul reduced their working hours to manage the illness. The work role also changed for Dave:

In some of the environments I’ve worked there’s definitely that testosterone culture where it’s often men in the senior roles so maybe there is that pressure that males are the dominant ones and are strong. This is mainly why I just had to change jobs [Dave, L130-135]

Paul was also aware and concerned with how his limited mobility deters him from participating in the male-orientated activities he previously engaged in. For example, drinking alcohol in a social setting is viewed as a dominant norm and cultural marker of masculinity (Herron et al., 2020):

I do definitely worry about the inability to meet the social expectations of being a male, like going to the pub [Paul, L26-28]

Later, Paul gave the impression that he feels he is not capable of carrying out mundane tasks, which had left him with a distinct level of self-hatred:

I can’t even like mow the lawn and that’s, well I feel anyway, a standard job that the male should do in the family [Paul, L61-62]

Likewise, Sam voiced the perceived cultural pressures to conform to the masculine stereotype:

In New Zealand there is that blokey culture and so men are very kind of reluctant to share when they’re struggling with their emotions and so I felt I could not share anything [Sam, L147-149]

Whilst there is evidence of adjustments being made in terms of lifestyle, participants still found it challenging using terms such as, ‘I can’t even mow the lawn’ and ‘I want to be the breadwinner’, indicating that receiving a diagnosis does not deter feelings associated with male incompetence.

Receiving support to deal with such challenges post-diagnosis can be pivotal for coping with a chronic illness, such as CFS; this is discussed in the next theme.

### Importance of support networks

Experiences of delegitimation are shown to be common for females with CFS (McManimen et al., 2019). Although not as widely studied, the work of Dickson et al. (2007) does suggest that it is likely to occur amongst males too. The current study revealed that delegitimation was common for participants in contexts beyond the clinical encounter:

My first supervisor who didn’t really support me in the way I would have liked was a male, however in my job after, my boss was a female and she was very supportive and even offered me to have regular breaks [Dave, L99-102]

Dave found more meaningful support having changed employers. For Sam, the delegitimation was nearer to home:

They (Sam’s family) aren’t that supportive really. They just simply don’t get the kind of day-to-day reality of it and I think because I don’t know any other males with it, I can’t get them to like tell my friends and family that they go through the same thing [Sam, L103-107]

Sam indicated that that sharing experiences with another male with CFS could help legitimise his own illness and be a way of encouraging his family to believe just how disabling the condition can be.

Furthermore, it appeared that finding support from other males was a challenge for some participants:

. . . .when I was diagnosed with depression, especially my male friends, they just thought I was being dramatic, attention seeking and exaggerating my worries. So that made me um really anxious to tell them (male friends) about my CFS as I just wasn’t confident anymore in myself [Paul, L19-23]

This could be attributed to the masculine norms previously discussed. As Sam reveals, participants were at times, reluctant to reach out for support:

In New Zealand there is that blokey culture and so men are very kind of reluctant to share when they’re struggling with their emotions and so I felt I could not share anything [Sam, L147-149]

However, receiving a diagnosis encouraged Simon to confide his worries surrounding masculinity threats with other males diagnosed with CFS:

Opening up to other males helped my mental health [Simon, L68-70]

Support groups and interventions for those diagnosed with CFS are not uncommon, although for males, they might not be viewed as accessible; Paul reveals a possibility as to why:

There are so many support groups out there for CFS which are targeted at mothers and often classes like yoga which I feel is female dominated. My male friends would definitely laugh at me for doing yoga anyway [laughs] [Paul, L35-38]

These extracts demonstrate the importance of encouraging men to talk to other men with CFS, as well as the development of support groups/interventions which promote gender equality.

## Discussion

The current research sought to gain a phenomenological understanding of the male experience of living with CFS, focusing on seeking medical advice, accepting a diagnosis and receiving support. Six themes emerged to demonstrate the participants’ experiences prior to, during and after obtaining their CFS diagnosis. Interviews highlighted that the symptoms experienced by males were consistent with the diagnostic criteria for CFS (NICE, 2021). Despite this, the group all had difficulties in gaining a diagnostic label, with masculine norms inhibiting recognition of symptoms and acceptance of a new life with a chronic illness. This was further compounded by a perceived lack of recognition of CFS as a legitimate health condition by the medical community.

The analysis illustrated that participants were initially unwilling to accept that they could be suffering from a potentially serious and long-term illness. Participants strongly assumed that HP’s do not offer as much sympathy for their physical suffering, as opposed to if they were a female. This reluctance to receive a formal diagnosis delayed help-seeking and was rooted in the perception that HPs would not take the symptoms seriously. This contradicts the findings by Clarke (1999) which proposed that men are more likely to be taken seriously by a HP. A meta-analysis of GP attitudes towards CFS found that approximately 39–44% of GPs did not accept CFS as a genuine, clinical condition (Pheby et al., 2021). Interestingly, it is common for GPs to state that they lacked confidence in their ability to diagnose or treat the condition (Pheby et al., 2021).

For these participants, gaining a formal CFS diagnosis often took many years, leading to feelings of frustration with the medical system. Despair was often compounded by a perception that symptoms were being falsely attributed to poor mental health. This reflects existing findings which reveal that many physicians are reluctant to characterise CFS as legitimate health condition (Thomas and Smith, 2005). Further to this, Rogers (2022) discussed that patients with CFS continue to feel the need to ‘fight for’ a diagnosis, despite its four-decade history of diagnostic entity. Ghali et al. (2022) demonstrated an association between illness duration and prognosis, where patients who experienced long diagnostic journeys were less likely to experience a spontaneous recovery.

Despite the reticence to believe their health concerns were related to a chronic condition, participants eventually accepted that obtaining medical advice was necessary due to symptoms disrupting day-to-day functioning and well-being. Appropriate management of CFS requires medical expertise and treatment varieties; once participants were diagnosed, they became desperate to source a GP with knowledge on CFS and its management. Experiencing a prolonged time between symptom onset and a medical diagnosis is likely to have impacted on participant recovery. For example, clinical outcomes of CFS patients demonstrated an association between illness duration and prognosis, where patients who experienced long diagnostic journeys were less likely to experience a spontaneous recovery (Ghali et al., 2022).

The current research adds to the growing body of literature on delegitimation (Cheshire et al., 2021; Dickson et al., 2007; McManimen et al., 2019). For instance, findings coincide with the challenges faced by men with other chronic illnesses, such as conforming to the idea of masculinity when suffering with rheumatoid arthritis (Flurey et al., 2018). Men here felt the need to ‘renegotiate’ their masculine identity to cope with their RA by enduring their pain to retain masculine activities (Flurey et al., 2018). Likewise, participants felt that the disabling nature of the condition meant that they could no longer adhere to, or identify with, many of the social representations of normative male behaviours, supporting findings by Wilde et al. (2019). Consequently, participants felt that by not conforming to stereotypical male activities, they would be perceived as not being truly masculine by others, such as close family members and friends. Some participants even blamed their loss of masculinity for experiencing the breakdown of relationships with friends.

Finally, the ability for participants to confide in their experiences to other males (with or without CFS), positively contributed towards illness acceptance and emotional comfort. This mirrors the findings from Wilde et al. (2019) where the role of support and understanding appeared integral to coping with CFS. Additionally, Wilde et al. (2019) reported that participants found social media-based support groups a beneficial source of CFS information. In the current study, however, men did not utilise support groups and instead, proposed the need for more widely available support groups targeted specially for men with CFS. To add, given that participants disclosed a lack of support from their workplace, so much so that one participant felt the need to change job, there is also an importance to educate employers and employees of the complexities of CFS and how support can be best offered.

The use of a qualitative approach to data collection and analysis offered several strengths and limitations. One strength is that IPA elicited rich, insightful accounts of CFS experiences from the participants themselves. Moreover, due to the nature of semi-structured interviews, all participants were able to talk freely at their own pace, an important factor to employ when considering that cognitive impairment represents a symptom of CFS (The M.E Association, 2020). A semi-structured style allowed for the participants to reflect on previous points discussed which aided depth of discussion. Likewise, as the interview guide was not fully structured, they were able to choose the direction of the interview themselves, which encouraged an informal and relaxed environment.

In terms of limitations of the study, the findings of this research are specific to the five participants interviewed and so results cannot be generalised to the wider CFS population. Whilst the study makes a significant contribution to an under-researched population, the sample was formed of predominantly white, partnered, heterosexual men. For example, men with CFS who identify as Black or Ethnic Minority (BME), and those living alone and/or in deprived circumstances may present different CFS experiences. Therefore, the results are suggestive, rather than conclusive but adds to the sparse literature specifically exploring the male experience of CFS. Regarding the participant recruitment process, a few individuals expressed their interest in taking part, but later decided that their intense fatigue would hinder their competence to participate. Furthermore, the interviews were only conducted at one specific point in time. Hence, it may have been useful to interview the same participants several months later to explore how the illness experience changed over time.

Overall, experiences documented in this paper can provide insight on how to improve and develop support for males with regards to negotiating their CFS journey. For instance, future research is needed to explore the effects of various CFS interventions. Magnezi et al. (2014) demonstrates that online health-related social networks for those with chronic illnesses can enhance patients’ self-efficacy by providing a space to discuss and comprehend their health expectations. Supporting this further, a study investigating online peer support groups for adults experiencing *Long COVID* found that groups offered a sense of symptom validation and reassurance (Day, 2022). Given that Long Covid poses similar characteristics as CFS in terms of symptoms, prognosis and aetiology, online networks targeting males with CFS are likely to be effective tools that support individuals to actively manage their health. Furthermore, implementing community-based men’s health programmes could be of benefit. For instance, the organisation ‘Men’s Shed’, mostly attended by males with mental health difficulties, provides a space for craftwork and social interaction and have been shown to foster a sense of belonging and social support facilitated by its socially acceptable and masculine environment (McGrath et al., 2022). Therefore, using a model akin to Men’s Shed specifically for those with physical health conditions, such as CFS, could be effective.

Developing a variety of interactive support groups could lower the prevalence of severe CFS cases by improving the rate of addressing symptoms, but it may also improve the alignment of current practice surrounding the NICE principles of care (NICE, 2021). In addition, establishing effective support mediums will enable GPs to signpost effective person-centred support for patients with CFS from the onset of their diagnosis.

## Conclusion

The research has highlighted the significance and mostly negative impact that hegemonic, masculine norms continue to elicit on illness perception and help-seeking behaviour in CFS. Therefore, there is a strong need to continue to legitimise CFS for male sufferers; HPs could contribute to this by implementing interventions which are tailored to the male experience. For example, designing support groups specifically for males which provide a non-judgemental environment to share their experiences. Future research could therefore explore the effectiveness of various online and in-person CFS support groups. Additionally, educating those in the workplace about CFS may not only encourage employers to implement appropriate support for staff with CFS, but it would also be a motivator for employees with CFS to be open about their condition.

## Supplemental Material

sj-docx-1-hpq-10.1177_13591053231186385 – Supplemental material for A phenomenological study on the lived experience of men with Chronic Fatigue Syndrome Supplemental material, sj-docx-1-hpq-10.1177_13591053231186385 for A phenomenological study on the lived experience of men with Chronic Fatigue Syndrome by Gracie Elizabeth Snell, Catherine Heidi Seage and Jenny Mercer in Journal of Health Psychology

sj-docx-2-hpq-10.1177_13591053231186385 – Supplemental material for A phenomenological study on the lived experience of men with Chronic Fatigue Syndrome Supplemental material, sj-docx-2-hpq-10.1177_13591053231186385 for A phenomenological study on the lived experience of men with Chronic Fatigue Syndrome by Gracie Elizabeth Snell, Catherine Heidi Seage and Jenny Mercer in Journal of Health Psychology

sj-docx-3-hpq-10.1177_13591053231186385 – Supplemental material for A phenomenological study on the lived experience of men with Chronic Fatigue Syndrome Supplemental material, sj-docx-3-hpq-10.1177_13591053231186385 for A phenomenological study on the lived experience of men with Chronic Fatigue Syndrome by Gracie Elizabeth Snell, Catherine Heidi Seage and Jenny Mercer in Journal of Health Psychology

sj-docx-4-hpq-10.1177_13591053231186385 – Supplemental material for A phenomenological study on the lived experience of men with Chronic Fatigue Syndrome Supplemental material, sj-docx-4-hpq-10.1177_13591053231186385 for A phenomenological study on the lived experience of men with Chronic Fatigue Syndrome by Gracie Elizabeth Snell, Catherine Heidi Seage and Jenny Mercer in Journal of Health Psychology

sj-docx-5-hpq-10.1177_13591053231186385 – Supplemental material for A phenomenological study on the lived experience of men with Chronic Fatigue Syndrome Supplemental material, sj-docx-5-hpq-10.1177_13591053231186385 for A phenomenological study on the lived experience of men with Chronic Fatigue Syndrome by Gracie Elizabeth Snell, Catherine Heidi Seage and Jenny Mercer in Journal of Health Psychology

sj-docx-6-hpq-10.1177_13591053231186385 – Supplemental material for A phenomenological study on the lived experience of men with Chronic Fatigue Syndrome Supplemental material, sj-docx-6-hpq-10.1177_13591053231186385 for A phenomenological study on the lived experience of men with Chronic Fatigue Syndrome by Gracie Elizabeth Snell, Catherine Heidi Seage and Jenny Mercer in Journal of Health Psychology

sj-docx-7-hpq-10.1177_13591053231186385 – Supplemental material for A phenomenological study on the lived experience of men with Chronic Fatigue Syndrome Supplemental material, sj-docx-7-hpq-10.1177_13591053231186385 for A phenomenological study on the lived experience of men with Chronic Fatigue Syndrome by Gracie Elizabeth Snell, Catherine Heidi Seage and Jenny Mercer in Journal of Health Psychology

sj-docx-8-hpq-10.1177_13591053231186385 – Supplemental material for A phenomenological study on the lived experience of men with Chronic Fatigue Syndrome Supplemental material, sj-docx-8-hpq-10.1177_13591053231186385 for A phenomenological study on the lived experience of men with Chronic Fatigue Syndrome by Gracie Elizabeth Snell, Catherine Heidi Seage and Jenny Mercer in Journal of Health Psychology

sj-docx-9-hpq-10.1177_13591053231186385 – Supplemental material for A phenomenological study on the lived experience of men with Chronic Fatigue Syndrome Supplemental material, sj-docx-9-hpq-10.1177_13591053231186385 for A phenomenological study on the lived experience of men with Chronic Fatigue Syndrome by Gracie Elizabeth Snell, Catherine Heidi Seage and Jenny Mercer in Journal of Health Psychology This article is distributed under the terms of the Creative Commons Attribution 4.0 License ( http://www.creativecommons.org/licenses/by/4.0/) which permits any use, reproduction and distribution of the work without further permission provided the original work is attributed as specified on the SAGE and Open Access pages ( https://us.sagepub.com/en-us/nam/open-access-at-sage).
