# Supplementary material for: A phenomenological study on the lived experience of men with Chronic Fatigue Syndrome
Source: J Health Psychol. 2023 Jul 17;29(3):225–37. doi: 10.1177/13591053231186385 (PMC10913334; doi:10.1177/13591053231186385)
Supplement: sj-docx-1-hpq-10.1177_13591053231186385 – Supplemental material for A phenomenological study on the lived experience of men with Chronic Fatigue Syndrome [file sj-docx-1-hpq-10.1177_13591053231186385.docx]

**Explanatory Memo**

Documents uploaded include:

1. Transcript analysis per participant (Research Data)
2. Emergent & Formulated themes (Supplementary Info)
3. Reaching theme consensus (Supplementary info)
4. Interview Schedule (Supplementary info)

The latest version of Otter was used; https://otter.ai/
