# Supplementary material for: A phenomenological study on the lived experience of men with Chronic Fatigue Syndrome
Source: J Health Psychol. 2023 Jul 17;29(3):225–37. doi: 10.1177/13591053231186385 (PMC10913334; doi:10.1177/13591053231186385)
Supplement: sj-docx-2-hpq-10.1177_13591053231186385 – Supplemental material for A phenomenological study on the lived experience of men with Chronic Fatigue Syndrome [file sj-docx-2-hpq-10.1177_13591053231186385.docx]

**Reaching Consensus**

**Yellow= SIMON Purple= SAM Turquoise= DAVE Red= PAUL Grey= TYLER**

***Fighting symptoms to maintain male competency.***

Before going to the doctors, I remember trying to just battle through it (L32-33).

I just tried to muscle my way through my hectic lifestyle (L14-15)

I didn’t want a diagnosis at first you see. I simply wanted to carry on playing football with the lads and going to the pub. I couldn’t say no because that would be embarrassing and they would just take the mick and tell me to “man-up” (L4-7).

Being a builder you have to be physically fit which I was. All my male collegues were so buff, so I tried to ignore my symptoms as I knew I had to keep training at the gym in order for me to keep my job like, like, I didn’t want to be seen as weak by them. (L45-49)

***Perception of healthcare inequality***

I could hardly stand up or even raise my arm in the air. Even at this point, I was still going to the doctors and they STILL said “well you have got here ok, so you must be absolutely fine. There’s nothing I can do” (L52-55)

one doctor point blank said to me that CFS doesn’t exist when I suggested to him that this could be what I am suffering from. Crazy [laughs] I even changed my GP surgery after that as I had just had enough (L73- 77)

I actually paid £2000 pound for MRIs (L83)…… I almost had hoped that the MIR scans would show up a brain tumour, just so then I would know why I was feeling like I do (86-90)

My GP, kind of accepted and treated me as if I had chronic fatigue, but I still think he probably, even 10 years later, probably thinks that it is mainly an element of psychological stuff going on, which is just simply not the case. (L35-38)

He originally said that I was too young to have M.E. But then eventually, they referred me to a hospital to see a chest specialist of all things (L30-32)

He called it ‘pseudo M.E’ and I was like no, this is not in my mind. I am not making it up or imagining the pain (L29-31)

There has been so much publicity on how males are not taken seriously when they admit how they are feeling, so this puts me off going. (L192-194).

I feel the health services are not male friendly. I feel that males are meant to be the ‘strong alpha male’ and so we are perhaps treated less sympathetically than females (L33-35)

***Challenges in accepting a new life***

Having a diagnosis gave me an answer and closure. (L-10)

I almost felt a sense of relief knowing that I wasn’t making it up and it was real. I could finally accept the new me. (L68-70)

‘I won’t let my M.E rule me’ is my motto that I tell myself. (L100-101)

I just kept doing things and then crashing. It was so bad and demoralising; I didn’t recognise myself anymore (L22-24)

I almost feel guilty, like I’ve stolen something from her by me being ill? I worry then that she will stop loving me. (L51-53)

Sometimes I feel invisible, and my old life has been snatched from me like some kind of punishment (L.119-120)

I just felt like a failure and a let-down to my family as soon as I heard him say he’s referring me to a CFS specialist, I felt that I was no longer the guy I used to be. (L94-96)

***Challenges to masculinity***

It’s my male pride that gets me…. my life has been based around work and doing as well as I can. And for that just to go It was really difficult. (L157-159).

the turmoil of it all drove me to feeling suicidal because I had real strong feelings of that. I just felt like a failure and a let-down to my family. (L180-176)

when I was diagnosed with depression, especially my male friends, they just thought I was being dramatic, attention seeking and exaggerating my worries. So that made me um really anxious to tell them about my CFS as I just wasn’t confident anymore in myself (L19-23)

I felt I need to be that male breadwinner, you know, I want to provide, want to kind of be a successful Father for my kid (L183-185)

in some of the environments I’ve worked there’s definitely that testosterone culture where it’s often

men in the senior roles so maybe there is that sense that males have to be the dominant ones and are strong. This is mainly why I just had to change jobs. (L130-135)

I do definitely worry about the inability to meet the social expectations of being a male, like going to the pub. (L26-28)

I can’t even like mow the lawn and that’s, well I feel anyway, a standard job that the male should do in the family. (L61-62)

In New Zealand there is that blokey culture and so men are very kind of reluctant to share when they're struggling with their emotions and so I felt I could not share anything. (L147-149).

***Importance of support networks***

My first supervisor who didn’t really support me in the way I would have liked was a male, however in my job after, my boss was a female and she was very supportive and even offered me to have regular breaks (L99-102)

They aren’t that supportive really. They just simply don't get the kind of day-to-day reality of it and I think because I don’t know any other males with it, I can’t get them to like tell my friends and family that they go through the same thing. (L103-107).

Even health professionals struggle to know what to say to me (L18)

I think the whole kind of assumption of competence as a male has kind of, to some extent, worked in my favour in terms of um my chronic illness not being as socially disabling as it might have been if I were a female. But then in other ways, I think it has kind of been difficult for me being a male due to the lack of recognition of non-physical suffering (169-175)
