# Supplementary material for: A phenomenological study on the lived experience of men with Chronic Fatigue Syndrome
Source: J Health Psychol. 2023 Jul 17;29(3):225–37. doi: 10.1177/13591053231186385 (PMC10913334; doi:10.1177/13591053231186385)
Supplement: sj-docx-3-hpq-10.1177_13591053231186385 – Supplemental material for A phenomenological study on the lived experience of men with Chronic Fatigue Syndrome [file sj-docx-3-hpq-10.1177_13591053231186385.docx]

**Emergent themes:**

***Experiences with the medical profession***

1. **Lack of support from HP’s**
2. **HP’s have limited knowledge on CFS**
3. **Distrust in medical profession**
4. **Frustration at HP’s**
5. **HP’s believe mental health is the cause**
6. **Misdiagnosis from HP’s**
7. **HP’s do not accept the illness**
8. **Lack of competence surrounding the diagnostic system**
9. **Negotiating a diagnosis**

***Journey to receiving a diagnosis***

1. **Determination to receive a diagnosis**
2. **Questions level of self-sanity**
3. **Trauma as a possible cause**
4. **Avoids symptoms**
5. **Received various medical tests**

***Receiving a diagnosis***

1. **Acceptance of self-identity**
2. **Relief to have an answer**
3. **Optimistic to have a diagnosis**
4. **Appreciation of what others think**
5. **Conscious of what others think**

**General views of CFS in Males**

1. **Stereotype for males to hide emotions**
2. **Men are reluctant to seek medical advice**
3. **Male competence causes illness to not be as socially disabling**
4. **Social norm that Men don’t speak up**
5. **Lack of male orientated medical support and therapy**

**Personal perception of the illness**

1. **Inability to succeed**
2. **Loss of male pride**
3. **Inability to meet physical expectations of being a male**
4. **Apprehensive of prognosis**
5. **Failure as a father figure**
6. **Loss of self-esteem**
7. **Loss of self-worth**
8. **Loss of friendships**
9. **Loss of personal control**
10. **Incompetence as a male**
11. **Pressure to conform to masculine roles**
12. **Inability to meet male social expectations**
13. **Inability to carry out stereotypical male job roles**
14. **Failure as a partner**

***Impact of CFS***

1. **Inability to perform mundane tasks**
2. **Condition affects work performance**
3. **Negative impact on social life**
4. **Limited in what physical activities can do**
5. **Loss of hobbies**
6. **Affects daily routine**
7. **Fatigue is the main burden**
8. **Life revolves around the condition**

***Coping Strategies***

1. **Support groups as a positive therapy**
2. **Hopeful with regards to Long-Covid**
3. **Awareness of symptom severity**
4. **The need to constantly plan**

**CFS support & understanding:**

1. **Lack of support from university**
2. **Lack of understanding by family**
3. **Lack of general recognition**
4. **Not taken seriously by others**
5. **Lack of understanding from peers**
6. **Lack of understanding by employers**
7. **Lack of understanding by work collegues**

**Formulated themes:**

1. **Hesitancy regarding one’s level of previous medical support gained**
2. **Awareness that avoidance behaviours caused health consequences**
3. **Inability to comply with the New Zealand culture**
4. **Gender differences in illness perception and treatment (discrimination)**
5. **The need to move job**
6. **Speculation surrounding gender differences regarding how one is supported by employers**
7. **Lack of control over life plans**
8. **Cannot meet expectations of a young person**
