# Supplementary material for: A phenomenological study on the lived experience of men with Chronic Fatigue Syndrome
Source: J Health Psychol. 2023 Jul 17;29(3):225–37. doi: 10.1177/13591053231186385 (PMC10913334; doi:10.1177/13591053231186385)
Supplement: sj-docx-4-hpq-10.1177_13591053231186385 – Supplemental material for A phenomenological study on the lived experience of men with Chronic Fatigue Syndrome [file sj-docx-4-hpq-10.1177_13591053231186385.docx]

**Interview Schedule**

SECTION A: Introduction

Hello there. Firstly, I would like to thank you for agreeing to participate in my research, it really is appreciated. My name is Gracie and I am currently studying a Masters degree in Health Psychology at Cardiff Metropolitan University. The aim of this interview is to gain an understanding of your personal experience with regards to living with CFS/ME. I just want to remind you of a couple of things before we begin:

- It is important that you know that although the information used will be discussed in my dissertation, your anonymity will be completely protected.
- I will be recording the interview to ensure a complete and accurate account of the information, but this will only be accessible to myself and my supervisory team.
- You will have the opportunity to review and amend the interview transcript before I use it in my research.
- If you have any questions for me during the interview then please ask. If you are uncomfortable discussing anything then please say and we can take a break, move on, or stop altogether. I would rather that you declined to comment than answer with what you think I want to hear.
- Take your time in answering questions – some of the things we’ll be talking about will have happened a long time ago so it is perfectly reasonable that you would take a few minutes to recall how you felt or responded to an event. There are no right or wrong answers in anything you say to me; it’s your life story after all!

**OK, before we begin, do you have any questions?**

***Press Record ***

SECTION B: Background of ppt

Firstly, I want to get to know a little about you.

1. **Tell me about yourself**
   *Probe: How old are you?
   Probe: Where do you live?*

*Probe: Who do you live with?*

1. **Please can you tell me about your hobbies and interests?** *Probe: Do you have a job?*

*Probe: Are you in education?*

*Probe: What do you like to do in your spare time?*

SECTION C: CFS/ME Diagnosis

This section of the interview addresses your experience with living with CFS/ME. Please try and think back to how you felt / thought at the time. Remember that the information you give me goes no further than here and the interview is not intended to catch you out in any way. If you are uncomfortable answering anything, or can’t remember, then just let me know and we’ll move on.

1. **Tell me about how you felt prior to receiving a diagnosis**

*Probe: What were your initial symptoms?*

*Probe: What made you seek help for your symptoms?*

*Probe: Had you heard of CFS/ME before/ know what the condition entails?*

*Probe: How did you feel- were you worried, scared, optimistic?*

*Probe: Were you aware of what your family/friends thought? If so, what did they think?*

1. **Tell me about your diagnosis**
   *Probe: When were you diagnosed?
   Probe: Were you diagnosed with a mild/moderate/severe form?*

*Probe: How long was the process for you to receive a diagnosis?*

*Probe: How did you go about receiving a diagnosis?*

*Probe: Doctor referral? Specialist?*

*Probe: Did you have any tests conducted for conditions other than CFS/ME?*

*Probe: Has the severity of your condition changed overtime?*

1. **How did you initially respond?**
   *Probe: What thoughts were running through your mind? Were you relieved/scared?
   Probe: What information did you at first receive and who from?*

***Probe: Experiences with health professionals*** *Probe: Did you have any particular coping strategies at this time- did they work?*

1. **How did your family, friends and employers initially respond?**
   *Probe: Did you have immediate support in place? If so, what?*

*Probe: Would you have liked more/less?*

1. ***How do your family, friends and employers respond to your condition now?****Probe: Do they accept/believe your condition?*

*Probe:* ***Stigma from society?***

1. **How has the condition impacted on your life?**

*Probe: What lifestyle choices have you had to make?
Probe: What is the most effective management strategy for you?*

That is all the questions I had planned. Is there anything from your perspective that we have not covered in relation to this topic that you would like to cover before we end?

**Thank you very much for your time.**

***Stop recording***
