# Supplementary material for: A phenomenological study on the lived experience of men with Chronic Fatigue Syndrome
Source: J Health Psychol. 2023 Jul 17;29(3):225–37. doi: 10.1177/13591053231186385 (PMC10913334; doi:10.1177/13591053231186385)
Supplement: sj-docx-5-hpq-10.1177_13591053231186385 – Supplemental material for A phenomenological study on the lived experience of men with Chronic Fatigue Syndrome [file sj-docx-5-hpq-10.1177_13591053231186385.docx]

**Participant- Tyler**

**Emerging Themes**

**Initial phrase**

****1 INT: Ok, so that is recording now. Before we start, have you any questions?

3 TYL: Nope, all good with me. I can hear you and see you so that is good [laughs]

Had a car crash in 1994

Diagnosed with epilepsy- possible cause?

Thought medication was causing the fatigue

GP ran tests- ECGs

Everything came back normal

Complained of lower back pain

Diagnosed with arthritis and spondylitis

Didn’t agree with diagnoses

Spoke to a specialist

Suffers with insomnia and muscle aches/pain

Tests demonstrated body inflammation

Unaware of what M.E is

Various diagnoses

Recognition of CFS from HP’s

4 INT: Yes, perfect [laughs] Okay, so um, so yeah, so just tell me about your experiences prior to getting the diagnosis. Did you know that it probably it was chronic fatigue?

7 TYL: Well I had a car crash in 1994 and I was diagnosed with epilepsy, maybe as a result of that I’m not sure. [INT: Okay]. And I was taking medication for that at the time and I thought that that was making me tired all the time, even though it stopped the seizures. So, I checked with the GP and they ran lots of tests like ECGs, but everything came back normal. But I just knew that it wasn’t side effects of the tablets, it was something else. So after still complaining of lower back pain and fatigue, I got diagnosed with arthritis. I also then [laughs] got diagnosed with spondylitis [INT: Ah, ok], but I just didn’t think that I came into that category so I had another chat with the specialist and they said that the things I’m describing, like I can’t sleep and the muscle aches and pain, he thought that sounded like M.E. So he ran some tests and found I had high …… in my blood which means that some part of my body is inflamed. Mine was something like 28 and I think normal is like 6. [INT: Yeah gosh]. So, the doctor said, this sounds very much like M.E and I said “M.E, what’s that”? [laughs] He said it’s also called CFS, but there’s no medication for it apart from the generic painkiller.

27 INT: Yeah, so did you sort of get the impression from the doctor that he thought it was more of a psychological thing?

29 TYL: Yeah definitely. He called it ‘pseudo M.E’ and I was like no, this is not in my mind. I am not making it up or imagining the pain. Before going to the doctors, I remember just trying to battle through it

Sense that CFS is not real

Lack of understanding of the condition by HP’s

Distrust towards some HP’s, but not all

Lack of support for males

Inability to carry out mundane tasks

Felt that doctors see CFS as a psychological illness and that one is making it up

Took eight years to get a diagnosis

Doctors thought symptoms were due to a lack of water intake

Treated properly in Cornwall, compared to Southampton

Provided with information on how to deal with CFS.

Relief

Feel CFS is more common in females

Lack of forums with males

Can’t do hobbies in which used to do

Have to go to bed straight after doing minimal activity

Likes to crochet

Brain seems to be on all the time

Suffers with sleeping problems

33 INT: Yeah, that's interesting. I've never heard of pseudo M.E.

34 TYL: It took me eight years to get diagnosed with M.E. I remember once the doctor said my symptoms are because I am not drinking enough water and I said “No, I drink 2 litres of water a day” [laughs]. [INT: Yeah]. I came down to Cornwall then however and it was a completely different scenario and I finally found like I was treated properly and listened to compared to when in Southampton. So I saw a specialist and he kind of gave me a set information on how to deal with um CFS.

43 INT: Ok yeah, that is good-

44 TYL: So, I then FINALLY got a proper diagnosis of CFS stroke M.E. Yeah, apparently, it's very rare for men to have it and females are more likely to have the condition due to their genetics. But I feel there's not very many forums for men to talk about M.E?

49 INT: Ok, let’s go back to that again later if you don’t mind as that is a good point. [TYL: Sure] So, it seems as if it has impacted on on your life significantly and, and, you know, you can't do the hobbies you would have would have liked to have done or maybe used to do.

54 TYL: Yeah, well I mean even when I go to cut the lawn, after that I would have to go straight to bed and rest. I’ve also been crocheting a lot recently, mainly because it rests my brain and calms me. I find I sleep better after doing it as well. But my brain seems to be on all the time, it just doesn’t switch off [laughs]. But yeah, I feel that there aren’t many mediums out there for men to discuss problems like sleeping etc.

Feels there aren’t many mediums out there for male support- groups are always targeted for women

Doctor said he was unique to be a male with the condition- reluctant to find another male to talk too

Grew up surrounded by women

Didn’t have a male model

Female led household made him stronger- Experienced women opening up about problems

Husband understands and accepts his condition

Friends and other family members don’t understand

Worries about telling friends and family about condition

Condition affects social life- worries that others think condition is an excuse

Deals with others

Speculation that there is a low CFS prevalence in males

Lack of male role model

Talking about emotions/physical illness relates to past experiences

Support from significant other

Lack of support from friends and family members

Negative impact on social life

63 INT: Ok, so why do you think that?

64 TYL: I think it's mainly because of support groups I feel are always targeted for women? I mean I can’t be the only male in the world who has CFS. Even my doctor told me that I am quite unique in the sense that I am male with this condition. So I think then, that just instantly made me feel that it will hard to find another male that is willing to discuss their experiences. You know?

71 INT: Yeah. So do you think that maybe males they don't speak out as much because they are perhaps perceived to be strong and and not be weak?

74 TYL: Yeah, I think so. I grew up surrounded by all women and so I didn’t really have a male role model as my Dad was out at sea a lot. But on the other hand, because I grew up in a female led household, I think this made me strong and I was always included when they would open up about their problems. So yeah, I am now aware of the importance of speaking out about your emotions etc. Luckily, my husband is very understanding with me and accepts my condition.

82 INT: Does the rest of your family and friends understand-

83 TYL: No, they don’t really. I get worried about telling them as I know what their reactions will be. For example, ‘Oh we all get tired in the afternoon’ or ‘oh it’s your age’. But having my diagnosis made me stronger in my mind. [INT: Yeah]. I know that my condition affects my social life as talking to other people just takes my energy away. I is also tiring just worrying about whether they think I am just using my condition as an excuse to not see them. [INT: Yeah]. So you are not only dealing with yourself, you are dealing with other people questioning you.

93 INT: Yes, I totally understand what you mean by that. I think it makes it harder when the condition just isn’t well known by the public. Also, you can’t see it [laughs] it’s not like a broken leg.

Feels can open up and share experiences with others with the condition

Strong minded

Talking about the condition helps

Feels there should be support groups just for males

Every aspect of life is affected

Gets pins and needles

Aware of more symptoms when know what others suffer with

True friends are the ones that have stuck by him

Lost more male friends than females

Feels invisible

Old life has been snatched like a punishment

Determination to continue with life as well as can

Variation of symptoms deters daily activity

Loss of belonging

97 TYL: Yes, I know. Even talking to you know with knowing that you suffer with the condition, I can really open up and share my experiences that I wouldn’t necessarily share with others as I know they just won’t understand! [INT: Yeah]. ‘I won’t let my M.E rule me’ is my motto that I tell myself.

102 INT: Oh, I love that. That is very true. A very good quote [TYL: Yeah, I think so].

104 TYL: Talking about the condition definitely makes me feel better and so I do think there should be some support groups made solely for males. Every aspect of my life affected, even driving, going to the shops etc. I get pins and needles a lot recently, symptoms seem to be ever changing and talking to people about the variety of symptoms is useful. Sometimes you don’t realise symptoms that you have, but when others mention it like online forums you think “oh yeah, I do have that actually, that explains it”.

113 INT: Yes, 100%. Ok well thank you so much for all of that. Have you got anything else you would like to add?

115 TYL: So with regards to the concept of male experiences, I feel that since my diagnosis my male friends that have stuck by me, they are my real friends, but I have definitely lost more male than female friends. Hmmm. But like yeah, sometimes I feel invisible, and my old life has been snatched from me like some kind of punishment.

121 INT: Aw, yeah. Yeah. I am sorry for you’re suffering. It is an awful illness. Well, I will just stop the recoding now so my recordings don’t get too big of a file size-
