# Supplementary material for: A phenomenological study on the lived experience of men with Chronic Fatigue Syndrome
Source: J Health Psychol. 2023 Jul 17;29(3):225–37. doi: 10.1177/13591053231186385 (PMC10913334; doi:10.1177/13591053231186385)
Supplement: sj-docx-6-hpq-10.1177_13591053231186385 – Supplemental material for A phenomenological study on the lived experience of men with Chronic Fatigue Syndrome [file sj-docx-6-hpq-10.1177_13591053231186385.docx]

**Participant- Simon**

1 INT: Thank you so much again for ehm taking part. It is much appreciated and it’s lovely to meet you. So, just to uh double check now that the recording has started, are you all ok to start and have no questions?

**Initial Key words**

**Emerging themes**

4 SIM: Yes, all good thanks.

Awareness of abnormal symptoms by comparison to peers

Suffering with trauma/grief is a possible cause

Condition negatively affects work performance

Suffers with brain fog/cognitive dysfunction

Diagnosed at the age of 40

Aware that something was not right from a teenager

Tired at the end of day

Didn’t know anyone else like it

Just want to sleep

Young, fit and healthy

Grief from the death of Father triggered symptoms

5 INT: Perfect. [pause] Ok so, if you don't mind just going back to when it all started really and how old were you when you were diagnosed?

8 SIM: I was diagnosed, well uh it must have been [pause] sorry, I’ve got to count as I'll get brain fog in a minute [laughs]

11 INT: No, honestly, please do not worry! Take your time.

12 SIM: I was diagnosed formally when I was 40, which was 5 years ago. However, I knew something was going on most of my whole life from my teenage years as things were just not adding up. I was working full-time as a builder, but I was um really tired at the end of the day, more so than anybody I ever knew who I was working with. I would go home and I’d just want to sleep as soon as I got in the door. [INT: Yeah] I felt this was not normal [pause] as I was young, fit and healthy.

20 INT: I see, yeah. Do you feel there is a specific reason for why you have developed the um condition, CFS?

22 SIM: Well [pause] around the time of my diagnosis my Dad passed away. That was when I stopped working.

24 INT: Oh, I am sorry to hear that-

25 SIM: I think there were a lot of other things going on as well, like the birth of my daughter. So, as you probably know, every life event, no matter how small it is, can uh make the CFS symptoms worse. I suffer quite a lot like with throat problems, like I often get like tonsillitis when I am run-down. I haven't had a bout in a while now actually, but early on I did, especially growing up in my teenage years, tonsilitis was a recurring thing for me a few times a year, as well as mouth ulcers I would get.

Significant life event worsened symptoms- birth of daughter

Regularly suffers with tonsillitis and mouth ulcers

Believed that poor mental health was the cause

Heightened flight and fight response

Doctors believe mental health is the cause and symptoms are made-up as still working

Everyday was a struggle

Lack of energy

Used to regularly train at the gym

Had a physical job as a builder

Could hardly stand up

Frustration at doctors as was not taken seriously- thought anti-depressants was causing symptoms

Significant life event is a symptom trigger

Experiences regular relapses

CFS was initially attributed to mental health by self & HP’s

Lack of recognition from health professionals

Fatigue is a burden

Loss of hobbies

Frustration at HP’S

CFS not perceived to be a serious condition by HP’S

34 INT: I see, ok. Yes, I often get tonsilitis too, a nasty thing!

35 SIM: myself and my wife initially put it down to my long suffering of anxiety and depression. My flight and fight response is so heightened. One possible symptom of M.E. I know that symptoms of depression can make you really fatigued. I feel that when I go to the doctors, they believe my mental health is what is causing my exhaustion and I uh sometimes feel that they think my anxiety means I am [pause] making how I feel up. I would go to the doctors and tell them that I feel unwell all the time, but because I tell them that I am still working they think I can’t be feeling that bad. Being a builder you have to be physically fit and healthy, which I was, all my other male collegues were so buff I tried to ignore my symptoms as I had to keep training at the gym in order for me um to keep job like, like, I didn’t want to be seen as weak by them.

50 INT: Hmmm.

51 SIM: So, uh it basically, my condition got to the stage where I could hardly stand up or even raise my arm in the air. Even at this point, I was still going to the doctors and they STILL said “well you have got here ok, so you must be absolutely fine.” My main doctor changed my anti-depressant medication so many times as he thought that my symptoms may be due to side effects of the medication.

58 INT: That must have been so frustrating for you. That’s interesting about the change of medication though. I wonder if many others have experienced the same.

Questioning level of self-sanity

Sense of low self-control

Relief that CFS is real/not mentally ill

Lack of CFS awareness from HP

Distrust in GP surgery

Determination to find an answer- took own initiative

Feared the worst, although hoped for the worst at the same time

Thought he was fantasying the symptoms

Worried about symptoms

Feeling a lack of control over the mind

Wife aware of symptoms

Never heard of the condition before

Felt relief to know that the condition is real and not made-up

Doctor said that CFS does not exist

Changed GP surgery

Frustration as no answers made the depression worse

Determined to find an answer so paid for private scans- MRIs

Scared that something was serious

Wanted to avoid the NHS waiting-lists

61 SIM: Hmm yes, that would be interesting to know. At one point I did even think maybe it could be me and I was simply fantasying all of my symptoms. So this caused me to research my symptoms as I started to get worried and felt out of control of my body. CFS did come up when I searched my main symptom …. me or my wife had never heard of the condition before, but symptoms that others were experiencing matched mine. I almost felt a sense of relief knowing that I wasn’t making it up and it was real. I could finally accept the new me. [laughs]

71 INT: Yeah, definitely! It’s a relief when you know you aren’t going crazy.

73 SIM: I remember though, one doctor point blank said to me that CFS doesn’t exist when I suggested to him that this could be what I am suffering from. Crazy [laughs] I even changed my GP surgery after that as I had just had enough and it was making my depression worse. So, that really made me determined to get to the bottom of what was going on with my uh body and so I paid for private scans. I was SO determined to find out what I was suffering from, well mainly in the hope that I could get treatment or at least, start to learn how to manage it. I actually paid £2000 pound for MRIs, because I had thought it may have been down to neurological abnormalities and was scared that something serious was going on with my health. [INT:Yeah gosh] The waiting list was so long you uh see to get the scan on the NHS, so I thought I would just pay for it myself. At one point, I almost had hoped that the scans would show up a brain tumour, just so then I would know why I was feeling like I do.

Hoped the scans would show up something

Deemed healthy by the doctors and a hypochondriac

Believed doctors thought he was a waste of time

Kept going back to the GP as no change in symptoms

Relieved when diagnosed as it gives a reason for the symptoms

Family and friends don’t know what the condition is

Helps to have diagnosis and reason for why lifestyle has to change

Lack of support/

appreciation from health professionals

Acceptance of self-identity

91 INT: Gosh and so did the scans show anything?

92 SIM: I was deemed ‘healthy’ by the doctors [laughs] and I got the impression they thought I was being a hypochondriac. I just felt like a failure and a let-down to my family as soon as I heard him say he’s referring me to a CFS specialist, I felt that I was no longer the guy I used to be.

97 INT: Oh my! [sighs]

100 SIM: Literally! I kept going back to my GP and reporting that I have had no change in my symptoms and eventually, a year after continuous trips to the doctors, I was formally diagnosed. Yeah. And … I know that there isn't necessarily a specific treatment that helps, but I think just having that diagnosis, it's just, you know, it's a reason for why you feel like you do. And even though a lot of people like my friends and family, they don't really know what the condition is, but if I just, if I can say that I have CFS, it sort of helps. I have a, what hopefully people think is a valid, reason for why I cancel plans etc.

111 INT: Yes, definitely. I know that um sometimes, having a label of a condition can cause difficulties, but I think with CFS, because you can’t physically like see it, having a medical like word that you can say does help, for sure.

115 SIM: hmmm yeah. Even though sometimes, it just takes up too much energy trying to explain what it is… and as a male, I find it really hard to tell people what my condition is. I don't have any friends anymore. You know, I had a lot of friends when I was building, but they don't see me anymore. I almost feel they have given up on me and just think I can’t physically do anything with them. Being a builder is also alpha male and so I felt that I could not live up to the expectations that they wanted me too both as a worker and as a friend. They often went to the gym after work, but I could never join them, despite wanting to!

Takes up too much energy explaining what CFS is

Finds it hard to talk about condition

Lost work friends

Feel friends have given up on him

Could not live up to the expectations of being a builder which is alpha male

Couldn’t join friends at the gym

Shy away from males

Can talk more openly to females

Hard to find someone that listens

Feel that friends think he is making up the symptoms of being lazy

Understands why others don’t get the illness

Lack of understanding about the condition

Appreciation for others

Distrust in medical profession

Inability to meet the physical expectations of being a Male

Loss of enjoyment in life- can’t do hobbies

Lack of general CFS understanding

Conscious of what others think

Appreciation of what others think

126 INT: Yeah. Do you think you find it harder to explain it to males more so than females then?

128 SIM: Yes, definitely. Yeah. 100%. Yeah, I'll shy away with males, whereas with a female, I feel I can talk more openly. And you know, I'm happy to talk sort of thing, but it's finding somebody that takes the time to listen because the majority of um people don't understand it and are not willing to. [INT: Yeah] And when I have told my male friends in the past, it's like, well, they want to go on and talk about something else instead. Again, I feel that they think I'm making it up sort of thing or I am being lazy. Hmmm.

137 INT: Yeah, I mean I do try and appreciate why people don’t understand it because again, you can’t like see it. And so, I DO kinda get why people just think, you know, they say to me, ‘get an early night’.

141 SIM: Yeah, yeah. And even, you know, years ago, before I got diagnosed, if somebody said they had M.E, I would have thought exactly the same. So I don't expect anything less. But I've even got relatives. I've got relatives, even a nurse. And I was trying a different treatment, I think it was to increase my oxygen levels and lower blood pressure etc. And she turned around and said to me, this is somebody in the medical profession, she told me, “go out for a run so you may sleep better” [laughs].

Distrust in the medical profession

Loss of Male Pride

Inability to succeed

Loss of self-esteem

Condition affects daily routine

Nurse told him to exercise to sleep better

Supportive wife

Wife understands

Struggles with his male pride

Worked all his life

Finds it difficult to come to terms with new life and not working

Old life was based around work and doing well

Had to deal with mental health problems

Feels inadequate

Believes that males should be the breadwinner and the strong one

Feels worthless

Daughter realises coping mechanisms

Has to sleep in the afternoon every day

150 INT: Yep, I definitely can relate to experiences like that [laughs]. So, do you have good support around you if you don’t mind me asking?

153 SIM: Yeah sure. Of course. My wife is helpful, yeah, she's really good. And really understanding which helps, even though I know you can’t properly understand it until you have lived with it… even for just an hour [laughs]. But I think it's my male pride that gets me because obviously, my life has been based around work and doing as well as I can. And for that to just go it was really difficult. I haven't worked since 2017. For me, you know, my life has been based around work and doing as well as I can. And for that just to go It was really difficult, but I had to almost let everything go. And that was hard, dealing with all those mental problems of not working and feeling inadequate, as well, as a male. There's always been always that thing of being the breadwinner or the ‘strong’ one of the house. So, yeah, you feel um quite worthless.

169 INT: Yeah, definitely. That must be tough when your life just suddenly changes like that.

171 SIM: Hmmm yeah.

172 INT: You say you have a daughter, is she aware of your condition?

174 SIM: Well, she is only six [laughs] but yeah, I think she's starting to realize that when it comes to midday or one o'clock in the afternoon, I've got to have a sleep. Basically, I won't last the whole day. And I think she understands that I’m often upstairs sleeping and so she probably has got used to it.

180 INT: Yeah.

Lack of confidence in being a Father

Apprehensive of the future in terms of condition prognosis

Lack of agreement in relation to advice given by counsellors

Difficulties establishing symptom severity

Feels a failure as a Dad

Sub-consciously knows that he is a good dad but wishes he could do more for daughter

Used to cycle

Finds it hard to reminisce on past life

Doesn’t like not knowing when or if the condition will get better

Had counselling in CBT sessions

Use of pacing

Feels that counsellors understand the condition.

Hard to pace energy

Feels it’s hard to save energy with a young daughter

181 SIM: But again, I do feel a failure as a dad. I'm always pushing to do more and more for her. I think, you know, on paper, I probably do do enough, but because I have to stop playing with her or whatever I feel bad. I think because I can remember what I used to do, I used to cycle a lot, I often look back on times like this and that almost makes things harder.

188 INT: Yeah. Hmmm. It is hard-

189 SIM: Also, I don't I don't like the feeling of not really knowing when or if it will sort of get better.

191 INT: Yeah, I know what you mean. And that does make it harder to know how far to push yourself. I know ‘pacing’ is a popular um treatment as such for this condition isn’t it.

194 SIM: Yes, absolutely. I mean, I’ve tried going to M.E support groups and I’ve had counselling and pacing comes up quite a lot, especially in CBT sessions. But even CBT sessions, it's so difficult. [sigh] I don't think even counsellors can properly understand the condition, you know. I think it's very hard to pace and see what you do and then pay, you don't know what energy you've got throughout the day to get used to it. And then they say, “Oh, don't do this. Don't do that. Save your energy”. But like, we've got a daughter, and I'm looking after that can drain my energy in half an hour.

204 INT: Yeah, definitely. And sometimes you do surprise yourself. Like, maybe going for a walk you didn't think that you could, but when you're actually out sometimes you think oh, I’m glad I did this as I do feel ok.

208 SIM: [laughs] Yes, literally! But then other times you think you're ok, and then you're not.

210 INT: Exactly that. YUP.

Appreciation for other CFS cases

Symptom management difficulties

Hopeful with regards to Long Covid

Misdiagnosis from medical professions

Frustration at the medical diagnostic system

211 SIM: Do help myself mentally, I try to keep busy and do jobs around the house each day. I can sort of see on the other hand, like how people just can't do anything and want to be in bed all day. But yeah, I couldn't do that; I like to keep going even though I'm probably not helping myself. It’s a constant battle between what you want to do and what you should be doing isn’t it!

Keeps busy as a coping mechanism for mental health

Understands why people with CFS want to be in bed all day

Finds it a constant battle to manage symptoms

Hopeful with the idea of ‘Long-Covid’ and CFS research/funding

Went for eye scans and a neurologist gave a diagnosis of

chronic migraine

Knew symptoms were not due to a migraine

Paid more money

Felt that gaining a diagnosis was like going around in a circle

218 INT: That is exactly what I always say to myself.

219 SIM: Perhaps one good thing about COVID is that hopefully, the idea of Long-Covid may lead to further research and possibly, um, more funding into CFS.

222 INT: I think exactly the same. Fingers crossed. So, you know, as soon as even next year it will be interesting to see if people are still suffering of long COVID.

225 SIM: Yeah, yeah.

226 INT: Yeah, or like two years later, because then that will really pinpoint the link between perhaps post viral fatigue and CFS. But, it's funny, you know, all the technology out there, you would have thought by now that they would have found the cause like-

131 SIM: Yeah, yeah, it's just frustrating. Oh yeah. I also went to eye scans and I went to see a neurologist and he diagnosed me with chronic migraine.

134 INT: Oh right

135 SIM: But at the time I uh, I mean, all the other symptoms, that can't just be from a migraine, you know? And then I went back to see him again, paid another 500 pounds. And he said, Oh, yeah, I think it could be M.E as well. I always thought, you know, my whole diagnosis just involved going around in a circle sort of things.

141 INT: I suppose you know, at least it was. I suppose you don't regret paying for all the scans because what if there was something serious you know.

Fear of speaking about condition to males

Positive experience with a clinical psychologist

Support groups allowed for acceptance and improved mental health

Doesn’t regret paying money for the scans

Had a specialist which visited his home

Encouraged to go to group meetings with other CFS sufferers

Reluctant to go

Worried about talking to males- shook him up

Took coverage to go

Suffers with Irritable bowel

Group of women were supportive

Group therapy helped

Had been assigned a clinical psychologist who specialises in CFS.

Found meetings helpful

Opening up helped mental health

144 SIM: Yeah, it's still a still a tricky one-

145 INT: So, do you uh have a specialist then that you go to at all?

147 SIM: Well, I had a specialist occupational health worker which came around visiting my home etc after I finally got uh the formal diagnosis. He encouraged me to go to a group meeting of other ME suffers.

151 INT: Oh. Right ok and how did you feel about that?

152 SIM: Well, I was reluctant to go at the time. And actually, [laughs] the first thing I asked was, are there any other males there? The answer was no. So that, that really shook me up again. And it took a lot of courage. And because of all my symptoms, were like, irritable bowel from like a stress, it was so hard to go. But I started to go and it's probably the best thing I did do, you know, although the groups involved all women, you know, and it was really hard for me, but actually, they were really accommodating. And it was actually the first time I spoke to people with the condition. Yeah, so that really helped. And then leading on to that, I had a clinical psychologist linked to me, she's a specialist in M.E. And she's really good. And she worked through past traumas I have encountered to work out whether they were possible triggers etc. So that was really helpful. I've been at that for over a year. And again, that was a big movement for me in opening up about my experiences. It also helped my mental health side of things.

170 INT: Yeah, no, that's, that's really good to hear.

171 SIM: Yeah. Yeah, it helped me open up because I could speak to other people. When I did talk about it, I wasn't so ashamed or embarrassed about the illness because I had meet met up with so many other people. It allowed me to finally accept my illness! I think up till that point. I was just in turmoil about it and feeling really low, you know, when but you know, and obviously, it'll be on tape and I'm not ashamed to say, but yeah, the turmoil of it all drove me to feeling suicidal because I had real strong feelings of that. I just felt like a failure and a let-down to my family.

Negative impact on mental health of not having a diagnosis

Inability to be the desired father figure and husband

Wasn’t ashamed or embarrassed

Allowed him to finally accept the illness

Before accepting it, he was in turmoil and felt really low- Drove him to feel suicidal

Felt like a failure and a let-down to the family

181 INT: OK. Well, uh, I think that is everything really. That is really helpful.

183 SIM: You are most welcome. If at anytime you want to ask me anything else then just email. I would also, uh, if able, I’d like to see the finishing project?

186 INT: Yes, of course! It’s due in September, so it will be around then.. all being well [laughs]

188 SIM: [Laughs] Well take care of yourself and best of luck.

189 INT: Thank you. Right. I will stop the recording now-

.
