# Supplementary material for: A phenomenological study on the lived experience of men with Chronic Fatigue Syndrome
Source: J Health Psychol. 2023 Jul 17;29(3):225–37. doi: 10.1177/13591053231186385 (PMC10913334; doi:10.1177/13591053231186385)
Supplement: sj-docx-7-hpq-10.1177_13591053231186385 – Supplemental material for A phenomenological study on the lived experience of men with Chronic Fatigue Syndrome [file sj-docx-7-hpq-10.1177_13591053231186385.docx]

**Participant- SAM**

1 INT: Okay, so that's recording, isn't it?

2 SAM: Yeah, yeah. Perfect.

**Initial phrases**

**Emerging themes**

****3 INT: Okay. So first of all, start back to how you sort of felt prior to receiving a diagnosis. Just talk about, you know, initial symptoms, what made you seek a diagnosis? That sort of thing. How long ago was it?

Student studying photography

Tried to battle through the symptoms

Drinks one litre of energy drinks to stay alert

Muscles his way through- unsure of what symptoms were

Symptoms didn’t go away

Had depression/stress as a teenager

Doctors assumed depression was cause

Prescribed anti-depressants

Saw a psychologist

Continued to try to figure out symptoms

Relief and optimistic

Hope

Relief

Optimistic

7 SAM: It was about a 11 years ago that I started experiencing extreme fatigue. And all of a sudden, I remember, I was driving to studying photography at the time, and I was driving to class and I just had to pull over to the side of the road, and I realised I just couldn't even get to class. And um, I was kind of just trying to battle through it. [INT: Yeah] I was drinking like one litre of energy drinks before every class to try and like, stay alert. I was just like, I just tried to muscle my way through my hectic lifestyle. And then just, it just didn't go away. Um. And so I've kind of been involved in the mental health system with issues like depression and stress as a teenager. [INT: Hmm mm] And so my doctors, of course, just assumed it was depression and gave me antidepressants. And I even saw a psychologist for a little bit through the public system. And they, [laughs] but let's say, I was trying to figure out what was going on. And my partner at the time was trying to figure it out as well, and I think it was her that came across chronic fatigue first. And when I remember kind of looking through the list of kind of symptoms, and what was involved, and I went, yeah, that's actually finally a sufficient, that actually explains kind of my experience. [INT: Yeah]. Yeah. relieved. And I was initially kind of optimistic because you really, we …. and they, you know, have various medical practitioners’ kind of have like a uhhh kind of um overall package that they suggest. Yeah. And I was quite optimistic that by changing some things, um my symptoms will go away. It's hard to know what counts in the formal diagnosis, because my GP, I think it's probably similar system to the UK, my GP, kind of accepted and treated me as if I had chronic fatigue, but I still think he probably even 10 years later, probably thinks that is probably an element of psychological stuff going on. [INT: Yeah]. So, so from him, I don't think I would necessarily even today, get a proper formal diagnosis. Yeah, I've got another doctor I see about meds and stuff, and they wrote me, oh I actually, I must have got a proper diagnosis from someone about six or seven years ago. But it's one of those things where it's getting an official diagnosis, and who's required to make that diagnosis. Or, like I literally 10 years later, and I'm in I'm in a in a registered social worker. So, you'd think I'd know these kind of things. I don't know. [laughs]

Avoidance behaviour

Relief and optimistic to have a diagnosis

Sense that health professionals do not accept the illness

Lack of competence surrounding the diagnostic system

Hesitancy regarding the level of previous medical support gained

Optimistic that treatment will deter symptoms

Thought GP though CFS is an element of psychological stuff

Diagnosed properly 6/7 years ago

Wary over who has the power to make that formal diagnosis-

Had various tests to rule out other diagnoses- Diagnosis of exclusion

Had every test under sun

Had blood tests

A sense that should have pushed for more specialists

48 INT: Yeah, that is frustrating. Did you have any other other tests? Because I know um here and like myself, I think it's sort of you've got to sort of have symptoms for at least six months to a year, and then other tests are done to rule out any other diseases etc. So all the blood tests and everything.

51 SAM: Yeah. Yeah. So, my GP and I have kind of gone through and tried to test for everything we possibly can. And it's yeah, it's a diagnosis of exclusion. So, yeah, I've had every test under the sun. I haven’t really seen specialists and perhaps I should have pushed more for other specialists. But I've had all the bloods and tests for other kinds of infectious diseases and all sorts of stuff and yet nothing has ever kind of come back to say that something is wrong or abnormal.

58 INT: Yeah. Do you think that by not pushing to to get a referral to a specialist, do you think it is because you, you maybe feel that they don't believe you?

61 SAM: Um. It was more so the lack of energy I had. Waiting for referrals and travel to clinics is just so time-consuming. Yeah. And I've been kind of I've been studying and doing other stuff. I've got a kid as well. So yeah, I kind of just manage my condition the best I can [INT:yeah]. But perhaps getting a specialist hasn't been perhaps as much of a priority as as it should have been.

Continues to study- avoidance behaviour

Further reflection on past actions regarding gaining professional support

Appreciation towards other CFS cases

Awareness of symptom severity level

Fatigue prevented seeing medical advice

Busy studying

Got a child

Manages condition the best he can

Feels should have seen more specialists

Condition is not highly severe- feels lucky for that

Energy envelope/window

Crashed many times and been bedbound

Thankful for not having fibromyalgia

Fatigue is the main symptom

Part-time PhD Student

68 INT: Yeah. Do you think that your condition has sort of got worse over time? Or does it come in, you know, waves of severity?

71 SAM: Yeah, so I definitely have never been as severe as kind of some people are, so I am quite lucky there. So, I've never been I've been bound for, like weeks.

74 INT: Yeah.

75 SAM: But it's um definitely related to how hard I push myself and staying within that energy kind of envelope/ window. So yeah, there have been many times where I've crashed and been bedbound for like a week or two. Yeah, so it can get quite bad. I don’t have the Fibromyalgia joint and other kind or pains, which I am SO thankful for. It’s mainly the fatigue for me.

82 INT: Ok, so I remember you saying that you're a part time PhD student? Is that because that's probably still more manageable doing part time, than full time-

85 SAM: Yeah. Yeah. So, I pushed myself to do full time study through my bachelor's. And I kind of just muscled my way through it. And I definitely harmed my health massively. Like I finished my degree six years ago, and I still think my health is still getting over it. [INT:Yeah]. But we had to do things like a full-time social workplace, which was 36 hours a week. And then we're expected to do coursework on top of it. And there were some minimal accommodations from the university, for me having chronic fatigue, but it just wasn't really taken seriously. [INT: Yeah]. And I didn't want to kind of put my life on hold for years and years and years to do it part time. So, I just decided to try and get through it. And as a result, I'm kind of I think I'm still paying the price of that.

Studying full-time harmed his health massively

Minimal accommodations from the university

Felt he wasn’t taken seriously by University staff

Tried to get through it but still paying the price

Always has to weigh up the pros and cons

The condition dictates his life

Supportive partner but not mother and siblings

Don’t get the day-to-day reality of it

Mum tried to offer complementary therapy

Aware that avoidance behaviour has caused health consequences

Lack of support from the university

Life revolves around the condition

Lack of understanding by family

Lack of trust in effectiveness of complementary therapies

98 INT: Yeah, it's tough, isn't it. You've got to sort of weigh up the pros, the positives and negatives of doing something.

100 SAM: Yup. Always weighing up the pros and cons of what I should do. It dictates my life.

102 INT: So, how do you, have you got support from family and friends? Do they sort of understand it? I know from myself, my mum suffered with it when she was younger. So that is quite nice to know this, you know, they believe you. But do you feel that you have support around you?

107 SAM: My partner is really supportive, really good. Completely kind of gets my energy level. We've been together for quite a long time, so she's terrific with it. Yeah. My parents, my mom and my kind of siblings, I don't think they get it. They simply don't get the kind of day-to-day reality of it. And just yesterday, my mom was trying to promote some kind of energy healing type crap [INT: [laughs]] and was like “I’ve got a friend who had something similar to you and they were cured using this” and I was like “yeah right” [laughs] So um, I think, like my doctor, I still think they probably think that it’s psychological.

118 INT: Yeah, I think you know, my friends that they, they are aware of it and they try to understand but I think you just can’t understand until you've experienced it yourself. And I have been thinking maybe with the coronavirus pandemic, that might lead to some insight, you know, long COVID and everything.

Lack of understanding

Hopeful in terms of long Covid-19 related research

Lack of appreciation by employment staff

Unconscious assumptions of male competence

Speculation over gender differences in terms of how CFS is perceived

Inability to comply with the New Zealand culture

Hope that people will start to believe the condition is real

He’s a staunch feminist

Believes the oppression of women systemically across society

Noone takes it seriously at the University

Assumption of competence in being a white male

Men can just do stuff and that they have to be strong

Discounted because a male

New Zealand culture causes men to be reluctant to share emotions

124 SAM: Yeah, so hopefully, people might start to believe the condition. Yeah and, um, I was thinking in terms of your research project, I’ve been kind of reflecting on masculinity and chronic fatigue. So, I'm a staunch feminist and I strongly believe the oppression of women systemically across society. So I'm not like trying to make any kind of say that women have it better because they certainly don't, but I think in terms of the chronic illness I have, I was thinking about it because I'm employed part time at the university and literally no one takes it seriously. I've said “look, I've got chronic fatigue, I see doctors and specialists about it, here's how it impacts my life etc”. And I've been wondering whether being a white male, there's that assumption of competence. And um whether if I were a female, they might go “ah, chronic illness, that is something which is real”. But they look at me and see a man and have those kind of unconscious assumptions that men can just do stuff and that they have to be strong. [INT:Yeah] Yeah, and so I'm wondering if the patriarchy is kind of leading to my illness being discounted in a way which would be different if I were a woman? Yeah, that's just pure speculation. But what I can say about masculinity and in New Zealand there is that blokey culture and men are very kind of reluctant to share when they're struggling with their emotions and so I felt I could not share anything.

150 INT: Yeah, that's what did prompt me to do this research. You know, it is known that males are less likely to seek help, especially for mental health. So, is that a reflection on a condition like this, that is perceived to be psychological?

Stereotype that men do not open up about emotions as this means a loss of masculinity

Inability to succeed to best standard

More open with it with my female friends

Believes there is a tough male culture in New Zealand

Highest male suicide rates in New Zealand

Blokey culture

Feels that supervisors accept that he can’t produce as much work as he would like, but don’t understand the condition

Use CFS when referring to the condition

154 SAM: Thinking about it, I've been much more open with it with my female friends, then my male friends, which probably does reflect the idea of the tough male culture, which is really prevalent in New Zealand, and we've got, New Zealand's got one of the highest male suicide and highest youth suicide rates in the world. We're very blokey culture. And so I think it's just not really done that men, open up. And so I think that that has been reflected in my experience that I haven't opened up to other guys about it.

162 INT: Yeah. Yeah. It is interesting.

163 SAM: Yeah, like, I feel like my supervisors, accept that I can't produce as much work as I would like, but they just don't understand what our chronic fatigue is or what it's like to kind of experience it.

167 INT: Yeah. And there's a lot of people that think, you know, well, just sleep more or maybe it's also that chronic fatigue it sort of just sounds like, you're just not sleeping, you know, you're you're staying up late. Yeah, it's funny, do you, if people ask you do you do you refer to it as chronic fatigue? or M.E? Chronic fatigue, it's obvious what it is then but M.E people aren't really, they're not sure. Are they-

173 SAM: I always use chronic fatigue just because it's a more accurate reflection of my experience. And I don't know if I have M.E because that refers to the swelling in the brain, right?

177 INT: Yeah. Maybe by the condition having 2 names that it is referred too accounts for the stigma because people are like “well which one is it”? etc.

180 SAM: Yeah definitely. So in terms of, like masculinity that I was reflecting on being a male is like unconsciously I am aware that this is like a stereotype and one that needs to be challenged. I felt I needed to be that male breadwinner and want to kind of provide, want to be a successful Father for my kidl. I'm sure it's the same for woman too, but I'm sure there are variations too, for in terms of being a male and experiencing that. And so there is that kind of feeling of like, because I've got a kid and I've got this, and things, I've kind of like letting the family down to some extent because I can’t be the primary earner and I can’t do some of the things that would kind of be expected. And like New Zealand kind of culture, people do a lot of activities outdoors I'm very limited in the degree to which I can do these things. The kind of things that guys would do around me like we go outdoor stuff or play sports, that sort of thing, it’s simply impossible with chronic fatigue. I wasn't sporty before my diagnosis, I was I was into other stuff, like camping and rock climbing. But I, I couldn't any longer go on protests or be involved in the animal rights activism I was doing.

Stereotype of masculinity

Sense of failure as a Father

Limited in what physical activities can do

Loss of previous hobbies

Loss of hobbies

Acceptance of symptom control

Aware that there is a stereotype of masculinity

Male breadwinner has to be successful

Feels he his letting the family down

Can’t be the primary earner

New Zealand- outdoor activities are common

Impossible to do sports

Used to camp, rock climb, animal campaigning

Watches a lot of TV and doing bonsai trees as a coping mechanism

200 INT: Yeah. Yeah. Which is a shame because well, you know, you still need to do your sort of hobbies and things that you enjoy for your mental health. So, it's keeping that sort of balance. [SAM: Yeah] So what are your preferred coping mechanisms to the fatigue?

155 SAM: Um, I do watch a lot of watching TV series. That's pretty much my go to for when my brain just has to rest. I've got a background of horticulture and I used to do community gardening and gardens and things like that. And I can't do that anymore because of the fatigue. So, I've now got into bonsai trees, so I can work on them at my own pace sat down in the garden [laughs]. And it's been really interesting, because I've gotten to it in the past year, and that is almost exclusively guys. And it's a really positive male community actually, like everyone is super chilled and supportive, it feels quite different. So that's been a really quite positive hobby to pick up and be involved in.

Involved in a supportive, male based activity

Pandemic and working from home has increased fatigue level

Intersection of masculinity and chronic illness- not considered before

Reflects on whether treatments are given differently in relation to gender

Condition viewed as socially disabling in females

Lack of recognition of non-physical suffering

Feels the invisible nature means illness is not noticed or recognised

Only females spoken to him about mental health

Found a supportive male community

Gender differences in illness perception and treatment

Male competence causes illness to not be as socially disabling

Lack of recognition for the illness

INT: Oh that is good. Yeah.

158 SAM: In terms of the pandemic, working from home has enhanced my fatigue as having my son and partner around causes more noise and stimuli etc. So, it is hard for me to rest.

160 INT: Yeah, I can understand that! Thank you for all of the points you have been making. Is there anything else you would like to add?

162 SAM: Yeah, it's been really interesting for me, thinking about the intersection of masculinity and chronic illness is something I hadn't really considered before. And, yeah, just kind of reflecting on whether I would have been seen or treated differently if I were a woman and what being a man has led to. And I think in some ways, I haven't been held back as much and that my boss kind of sees me and ignores the fact that I've got a chronic illness. And so, I think the whole kind of assumption of competence as a male has kind of, to some extent, worked in my favour in terms of um my chronic illness not being as socially disabling as it might have been if I were a female. But then in other ways, I think it has kind of been difficult for me being a male due to the lack of recognition of non-physical suffering. I think the invisible nature of the illness means it is not noticed or recognised. Also, I've had several female friends have opened up to me about their mental health struggles. I don't know if a single man in my life is ever opened up about the fact that they're mentally struggling and I think this would be the same with chronic fatigue. I have certainly have only told females about my condition and no males. [INT: Yeah]. I mean males have told me that life is tough for them, but they never go into details about their suffering etc.

Inability to do desired physical activities

The need to constantly forward plan as a symptom management strategy

Stereotype for males to hide emotions

Not taken seriously by others

Men are reluctant to seek medical advice

Male competence causes reluctance to seek help

Only told males about his condition

Partner understands

Can’t do physical things

Has to be careful about what activities he does

Has to work out what to say to his son

Believes that autoimmune disorders are more common in women

Hormones

Females are easily emotional and are encouraged more to talk about their emotions

Contraception

Feels that males are not taken seriously

Believes that CFS is female dominated

‘man-up’- causes reluctance

Afraid to go to the doctors as worry what friends would think

177 INT: Yeah.

178 SAM: My partner understands that I can’t do physical things but like my son, he wants to go mountain biking, and all that kind of stuff. Stuff which I have to be very careful about what I do. I don’t want to say I’ll do something with him and then when it gets to the time I can’t do it because I’m too tired. I feel I always have got to work out what you’re going to say.

182 INT: So, just one more question ok. What are your thoughts on why CFS is more prevalent in females than males?

184 SAM: Well, I think autoimmune disorders are more common in women, so maybe it is due to biological factors? Perhaps it’s due to hormones as well. I know that females do get easily emotional when they go onto hormone contraception, so perhaps they find it easier to talk about their emotions or people can physically see they are in distress and so are encouraged to talk about their symptoms more so than males because they are more inclined to hide their symptoms? There has been so much publicity on how makes are not taken seriously when they admit how they are feeling, so this puts me off going. I actually have a male friend now who suffers with depression, but whenever I ask how he is, he shrugs it off and says he is fine. I also think that CFS is ‘female dominated’ and that men are often less likely to seek medical help for any condition unless it is life-threatening. [INT:Yeah] Also, the phrase of “man up” often make men reluctant to seek a diagnosis. I was afraid to go to the doctors about my symptoms as I was worried that my male friends would take fun out of me and tell me to stop moaning… but yeah, my family aren’t that supportive really. They just simply don't get the kind of day-to-day reality of it and I think because I don’t know any other males with it, I can’t get them to like tell my friends and family that they go through the same thing.

108 INT: Thank you they are good points, well that’s everything I have to say. We’ve covered a lot.

110 SAM: Sure. You're welcome to flick me through an email if you have any other questions or if you want to set up a follow up interview, I'm open to that. I don't know what your ethics allows you to do. But just let me know if you do want to, if you've got anything comes to mind, particularly later on in the project, you might go hang on a second, I wish I'd asked that. So, feel free to get in touch.

117 INT: Oh, thank you [NAME]. I really, really do appreciate it. I am struggling a little bit to get participants so thank you. Right I’ll stop the recording now-
