# Supplementary material for: A phenomenological study on the lived experience of men with Chronic Fatigue Syndrome
Source: J Health Psychol. 2023 Jul 17;29(3):225–37. doi: 10.1177/13591053231186385 (PMC10913334; doi:10.1177/13591053231186385)
Supplement: sj-docx-8-hpq-10.1177_13591053231186385 – Supplemental material for A phenomenological study on the lived experience of men with Chronic Fatigue Syndrome [file sj-docx-8-hpq-10.1177_13591053231186385.docx]

**Participant- Paul**

**Initial phrases**

****1 INT: Ok, so that is recording-

**Emerging Themes**

2 PAU: Perfect

The need to establish personal identity

Limited CFS knowledge in general

Lack of understanding from male friends and health professionals

Inability to meet male social expectations

Diagnosed 8 years ago

Relief to get diagnosis as took 4 years to work out what was going on

Diagnosis gave answer and closure

No awareness of CFS- Feel that people pretend to know what CFS is

Friends roll their eyes

Health professionals struggle to know what to say

Friends thought being dramatic when diagnosed with depression

Anxious to open up about CFS

Can’t go to the pub with male friends

3 INT: Ok so, first of all, tell me about your diagnosis.

4 PAU: I didn’t want a diagnosis at first you see. I simply wanted to carry on playing football with the lads and going to the pub, I couldn’t say no because that would be embarrassing, and they would just take the mick and tell me to “man-up”. Ok, so I was formally diagnosed about 8 years ago. It was such a relief to get a diagnosis as it took me roughly 4 years to work it out. Having a diagnosis gave me an answer and closure. [INT: Yeah] I think it took me so long to get a diagnosis because there just wasn’t awareness or knowledge on the CFS. Even now, when you tell people you have CFS, almost everyone is like “what is that?” Even when they do say “oh yeah”, I always feel that they are just pretending to know just to be nice. Although it was a relief, my friends roll their eyes when I tell them what I suffer with and even health professionals struggle to know what to say to me. [INT: Yeah] It was the same when I was diagnosed with depression, especially my male friends, they just thought I was being dramatic, attention seeking and exaggerating my worries. So that made me um really anxious to tell them about my CFS as I just wasn’t confidence anymore in myself, you know, join them at the pub for a pint. [INT: Yeah] Like I can’t go to the pub because alcohol just worsens my symptoms. So, I do definitely worry about the inability to meet the social expectations of being a male, like going to the pub.

29 INT: Ok. That’s interesting about urh your male friends not really understanding and you and worrying-

Worried about seeking help

Male services are not male friendly

Feel that males are meant to be strong and treated less sympathetically than females

CFS targeted at mothers

Yoga classes are female dominated

Worries about what male friends think

Supportive wife

CFS hindered relationship

Been trying for a second child for a while but anxiety medication lowers libido

Feels like a failure/guilty- worry won’t be loved by wife

Can’t watch son play rugby as gets achy legs and painful joints

Compares to other Dads

Can’t mow the lawn which is a standard job for males- stereotype

Need for male competence

Lack of male orientated medical support and therapy

Embarrassment to partake in male-related activities

Failure as a Father

Loss of self-worth

Inability to carry out stereotypical male job roles

Father figure comparison

31 PAU: Yeah. Yeah. I was also worried about seeking help initially as I feel the health services are not male friendly. I feel that males are meant to be the ‘strong alpha male’ and so we are perhaps treated less sympathetically than females? There are so many support groups out there for CFS which are targeted at mothers and often classes like yoga which I feel is female dominated. My male friends would definitely laugh at me for doing yoga anyway [laughs]

39 INT: [laughs] I mean, I shouldn’t laugh as that is such a shame as I know how much yoga can help to ease symptoms.

42 PAU: Yeah. My I do do it actually wife my wife, but of course don’t tell my male friends [laughs]

44 INT: Is your wife supportive?

45 PAU: Yes, she is. Although, I hope I can say this on record [laughs] but my um relationship with her has hindered slightly since I was diagnosed [INT: oh right-] Yeah. Yeah, well we have been trying for a second child for a while now, but the medication I take for my anxiety causes side effects that lowers my libido, on top of my fatigue. So, I always feel so bad that I don’t give her the intimacy she wants. I almost feel guilty, like I’ve stolen something from her by me being ill? I worry then that she will stop loving me. I feel like a failure as I can’t provide a big family that me and my wife would love. I do have a son, (NAME) but even that, I can’t go and watch him play rugby as standing up outside for a long time sets off my achy legs and painful joints. [INT: Yeah] So yeah, that really sucks because I know that all of his friends, all of their Dads go and watch their sons play rugby. My wife doesn’t understand rugby so she’d be useless! [laughs] Also, I can’t even like mow the lawn and that’s, well I feel anyway, a standard job that the male should do in the family. Maybe this is an old-fashioned way like in terms of when the men went to war etc, but males are seen as the main earner of the family and I haven’t worked now for years.

Failure as a husband

Inability to perform mundane tasks

Condition affects work performance/

ability

Lack of understanding from employer

Acceptance difficulties in terms of lifestyle changes

Lack of control over life plans

Feels males are the main earner

Hasn’t worked for years

Crippling fatigue and brain fog

Can’t help the wife out with food shopping

Suffers with memory difficulties

Used to work in a school as a teaching assistant full-time

Used to train in the gym every night

Had to move to part-time but that was a struggle

Boss was supportive

Suffered with glandular fever- possible cause as didn’t recover

Long-term fatigue

Used to be so active

Took a while to accept illness and that his old life was no longer

Planned to go into the army

Couldn’t pursue career aim

66 INT: Yeah. Yeah, I get what you mean.

67 PAU: I just can’t work due to my crippling fatigue and brain fog. That reminds me, like I can’t even help my wife out and do the food shopping as I forget like what brands to buy and things we need.

71 INT: Aw yeah, that must be tough. Where did you work before?

73 PAU: Well, I used to work in a school as a teaching assistant. I was working full-term, as well as training at the gym every night. But then when I gradually started to become ill and notice the CFS symptoms I went to part-time, but then even that was a um struggle. I mean I literally told my boss that I either go part-time or nothing at all like as I knew he wouldn’t understand why I need time to rest [laughs]

80 INT: Yeah ok. Do you think there was any particular reason for why you developed the condition?

82 PAU: Um, well I did suffer with glandular fever when I was in my early 20s, so I feel maybe that was it? I just didn’t recover and the fatigue just stayed with me. But I always used to be so active, so it took a while for me to accept that my old life was no longer. I was planning on going in to the army, so I really struggled to come to terms with the fact I couldn’t pursue my career aim.

89 INT: Yeah, I bet. [PAU: Yeah] Ok, well is there anything else you would like to add about your overall experiences?

91 PAU: Nope, that’s it for me.

90 INT: OK, well thank you so so much. I really appreciate it-
