# Supplementary material for: A phenomenological study on the lived experience of men with Chronic Fatigue Syndrome
Source: J Health Psychol. 2023 Jul 17;29(3):225–37. doi: 10.1177/13591053231186385 (PMC10913334; doi:10.1177/13591053231186385)
Supplement: sj-docx-9-hpq-10.1177_13591053231186385 – Supplemental material for A phenomenological study on the lived experience of men with Chronic Fatigue Syndrome [file sj-docx-9-hpq-10.1177_13591053231186385.docx]

**Participant- Dave**

1 INT: OK, so that is recording. So, just before we start, have you any questions?

**Emerging Themes**

**Initial phrases**

****3 DAV: Nope, all good. Fire away [laughs]

Avoidance behaviour

Loss of self-esteem and self-worth

4 INT: Ok, so when did your condition develop?

Symptoms started at age 16

Used to play football, row and run

Initially thought symptoms were the flu/a cold

Tried to carry on

Crashed on the sofa after rowing

Doctors thought it may be glandular fever

Swollen glands and constantly fatigued

Didn’t know how to manage it

Used to leading a busy life

Constantly crashing

Demoralising

Kept going back to the GP

Doctor said too young to have M.E

5 DAV: Ok so, it started when I was about 16. Before that I did lots of sports like football, rowing and running and then I got ill and um I think initially, I thought the symptoms was the flu or a cold or something, so I just tried to carry on doing what I was doing. And I remember quite vividly going to this rowing race and trying to do it, but literally could not and came back and just crashed on the sofa and this was something I had not felt before. [INT:Yeah]. So I went to the doctors and they thought it may have been glandular fever and I did some tests. My doctor told me to rest and it will go in a few weeks, but it just didn’t. My glands were so swollen and I was constantly fatigued but I was aware glandular fever can last a while so I gave it a few more weeks. But during this time, because I didn’t really know what was going or how to manage it- [INT:Yeah] so I was at secondary school and I was used to doing, or leading a life that was quite different, like doing lots of activities etc and because I didn’t know how to manage it, I just kept doing things and then crashing and doing things and then crashing. It was so bad and demoralising, but I didn’t really know what I was doing wrong at the time. So basically, we we kept going back to the GP, but to be honest there wasn’t a huge awareness at the time of what M.E was- [INT:Yeah] so I remember at one point, my Mum, who works with the NHS explained to a colleague what I was going through and she said “well could it be M.E” so my Mum phoned the doctor and asked him whether it could be M.E, but he originally said that I was too young to have M.E. But then eventually, they referred me to a hospital to see a chest specialist of all things, but they did lots of tests to rule out over things etc. So I first had symptoms when I was 16, but I was diagnosed when I was 17. When I had my diagnosis and knew more about it, it helped me a lot to understand my condition [INT:Yeah] It was definitely worse when I was younger, but I think that is because know I am more aware and I can recognise early signs of a crash for instance.

Lack of understanding by HP’s

CFS awareness and recognition lowers condition severity

Inability to continue with previous hobbies

Lack of understanding from peers

Loss of friendships

Eventually referred to see a chest specialist

Had lots of tests to rule other illnesses out

Diagnosed when 17

Diagnosis helped understanding

Can recognise early signs of crashing

Football team would not understand

Thought condition was seen as an excuse

Was able to move around and go to school

Lack of appreciation and understanding

Lost male friends because of it

40 INT: Yeah definitely. In some ways it is a relief to have a diagnosis. It’s an answer to people for your behaviour.

42 DAV: Yeah. Yeah. 100% and and I remember telling like the football club that I can’t play anymore as I’m unwell with this condition. They would say “ok, well are you free to play next weekend” and I was like it’s not something that just goes away in a week [laughs]. So yeah, there was a lack of really um understanding and definitely, from some quarters it was definitely seen as an excuse I guess as they didn’t view it as a serious thing. Like if I had something like diabetes or a broken leg where you can see it, then I think they would have taken it more seriously instead of “well you look fine and you used to play football all the time” [INT:Yeah] Um, so and and I guess the other thing is that sometimes people get it to different severities, like I was able to go to school and move around and I think they thought that if you can walk to lessons then why can’t you do other things like football. So yeah, there was a lack of appreciation and understanding, which was hard. Yeah I did loose some male friends because of it.

62 INT: Yeah, that must have been tough, especially like watching them playing football because it’s not as if you didn’t want to do it anymore, you would have if you could have you know? So it’s almost like the condition prevents you from doing things that you enjoy doing which really, it really sucks.

Declared condition at University

No understanding

Lack of awareness of what support is needed

First job was in a law firm

Saw an occupational therapist

Capped hours

Supervisors weren’t on board with accommodations

Feel employers see leaving early as being lazy

Lack of understanding

Lack of understanding from University staff

Lack of understanding by employers/work collegues

68 DAV: I remember when I went to University I declared the condition and I remember they made me meet somebody and again there wasn’t an understanding and so said “so you don’t any extra help or anything then” and I said well “yeah I could do with some” and then they literally took it to the other extreme and said “ok, so we will have someone sit next to you in lessons so they can write for you” and it was like “no, no I don’t need that either” [laughs]

75 INT: How is your work now?

76 DAV: Well the first job I went into after Uni was the law firm and they have a reputation of working the employees really hard. So I got the job first and then I told them I have this condition and originally they made me come in and see them and see an occupational health therapist and they initially agreed that they would cap my hours as some trainees work until 2am in the morning, which I physically could just not do. So we had this agree to cap hours, but in practice it was really really hard because it’s all well in having capped hours, but you need supervisors to be on board with it because if they give you lots of work or didn’t explain to other members of the team, then they just think I am being lazy by leaving early. So again, I feel that is another example of how there is such a lack of understanding there. [INT:Yeah].

90 DAV: It was actually during the law firm that I had a bad relapse which I hadn’t had in a while. This was about in 2015 and I went back to hospital in London then to recover. The specialists did try many different things, like vitamin D supplements, but that didn’t work. I had graded exercise therapy and the specialists would speak to occupational therapists. So, all in all, things did start to improve. But ultimately, I felt I did have to move to a job where there was less expectation on doing long shifts, because it just felt difficult always having to explain it to people and people judge and misinterpret it- [INT: and that’s tiring in itself isn’t it] Yeah it is. Interestingly, my first supervisor who didn’t really support me in the way I would have liked was a male, however in my job after, my boss was a female and she was very supportive and even offered me to have regular breaks and to start at a later time in the morning.

Had a bad relapse in 2015

Went to London hospital to recover

Had vitamin D supplements

Had graded exercise therapy

Had to move job to where there was less expectations

Finds it difficult to explain the condition

Others judge and misinterpret it

Male boss didn’t provide nay support but female boss did

Appreciates others trying to understand

People accommodate you as if you are severely disabled

Doesn’t find it easy talking to collegues about it

Don’t want to be defined as the guy with M.E

High expectations that trainees should be capable

Variations in treatments

Lack of understanding meant had to move job

Gender differences regarding how one is supported by employers

Appreciation for others in their understanding

Maintenance of male pride

Lack of understanding

Cannot meet expectations of a young person

106 INT: Oh ok, so that is interesting in terms of the differences in males and females!

108 DAV: Yeah, but you’re right, it’s not something that when you explain it to people they know what it means straight away [INT:Yeah] and I do appreciate people trying to understand. Sometimes people think they are helping but sometimes they make things worse and almost accommodate you as if you are severely disabled. I don’t find it the easiest talking about it to collegues in the workplace as you don’t want them to define you by your condition or remember you as the guy that has M.E [INT:Yeah]. But then you kind of, if you don’t tell people, you can’t expect them to act in a supportive way to you. And I think there is that expectation with a trainee that “oh you look young and healthy, and you should be able to work these long hours”.

122 INT: Yeah. So, do you think there is a difference in how people perceive the condition in males, compared to females?

Feel that men culturally don’t speak about ill health- a sign of weakness

Social norm

Testosterone culture in the workplace

Males are under pressure to be strong

Doesn’t know anyone with the condition

Feels there are a lack of support groups

Hope with Long-Covid

Reflects on the idea of Yuppie Flu- Symptoms were due to over working

M.E used to be a taboo subject

People were thought to be crazy

Covid may change perception/ attitudes of CFS severity/ prevalence

Social norm that Men don’t speak up

Male competence

Pressure to conform to masculine roles

Long-Covid- change in CFS attitudes

Long-term lack of CFS understanding

Associations of CFS and being mentally ill created taboo societal beliefs

125 DAV: Yeah, that’s a good question. I mean I don’t know how I would feel if I was a women myself, but I think yeah, culturally, I think Men don’t speak up because it’s a sign of weakness if someone is struggling with their health. So, maybe there’s that social norm that men just don’t speak about personal matters. And certainly, in some of the environments I’ve worked there’s definitely that testosterone culture where it’s often men in the senior roles so maybe there is that pressure that males are the dominant ones and are strong. This is mainly why I just had to change jobs. I don’t know anyone with the condition, so it is hard to analyse how people go about telling people about their experiences or simply managing the condition. That may be because there are a lack of support groups out there, I don’t know. [INT: Yeah]. Hopefully, the long-covid health condition that has been discovered will hopefully encourage more awareness on this condition and then people may realise that they are suffering with this condition. It’s like when they used to label M.E as ‘Yuppie Flu’ back in the day and these people were the highflyers as such, working long hours etc and people simply said that the M.E symptoms were due to overworking and burning yourself out.

147 INT: Yeah, that is a good point yeah-

148 DAV: Yeah. And I think with the long-covid, M.E used to always be such a taboo subject and no-one wanted to talk about it as people who had it were thought of has being ‘crazy’ or ‘mentally ill’, but now they are almost saying that the long-covid is worse than actually having Covid [INT: Absolutely] and so perhaps this will change people’s attitudes towards it and also, people will start to realise that it is actually a real illness!

156 INT: Ok, well thank you for that. Have you got anything else to say? I think we’ve covered pretty much everything, and I don’t want to take up too much of your time!

159 DAV: Aw, no, it’s fine. But yes, I don’t have anymore to say.

160 INT: Ok well I will stop the recoding now-

**Participant 2 Paul**

**Initial phrases**

****1 INT: Ok, so that is recording-

**Emerging Themes**

2 PAU: Perfect

The need to establish personal identity

Limited CFS knowledge in general

Lack of understanding from male friends and health professionals

Inability to meet male social expectations

Diagnosed 8 years ago

Relief to get diagnosis as took 4 years to work out what was going on

Diagnosis gave answer and closure

No awareness of CFS- Feel that people pretend to know what CFS is

Friends roll their eyes

Health professionals struggle to know what to say

Friends thought being dramatic when diagnosed with depression

Anxious to open up about CFS

Can’t go to the pub with male friends

3 INT: Ok so, first of all, tell me about your diagnosis.

4 PAU: I didn’t want a diagnosis at first you see. I simply wanted to carry on playing football with the lads and going to the pub, I couldn’t say no because that would be embarrassing, and they would just take the mick and tell me to “man-up”. Ok, so I was formally diagnosed about 8 years ago. It was such a relief to get a diagnosis as it took me roughly 4 years to work it out. Having a diagnosis gave me an answer and closure. [INT: Yeah] I think it took me so long to get a diagnosis because there just wasn’t awareness or knowledge on the CFS. Even now, when you tell people you have CFS, almost everyone is like “what is that?” Even when they do say “oh yeah”, I always feel that they are just pretending to know just to be nice. Although it was a relief, my friends roll their eyes when I tell them what I suffer with and even health professionals struggle to know what to say to me. [INT: Yeah] It was the same when I was diagnosed with depression, especially my male friends, they just thought I was being dramatic, attention seeking and exaggerating my worries. So that made me um really anxious to tell them about my CFS as I just wasn’t confidence anymore in myself, you know, join them at the pub for a pint. [INT: Yeah] Like I can’t go to the pub because alcohol just worsens my symptoms. So, I do definitely worry about the inability to meet the social expectations of being a male, like going to the pub.

29 INT: Ok. That’s interesting about urh your male friends not really understanding and you and worrying-

Worried about seeking help

Male services are not male friendly

Feel that males are meant to be strong and treated less sympathetically than females

CFS targeted at mothers

Yoga classes are female dominated

Worries about what male friends think

Supportive wife

CFS hindered relationship

Been trying for a second child for a while but anxiety medication lowers libido

Feels like a failure/guilty- worry won’t be loved by wife

Can’t watch son play rugby as gets achy legs and painful joints

Compares to other Dads

Can’t mow the lawn which is a standard job for males- stereotype

Need for male competence

Lack of male orientated medical support and therapy

Embarrassment to partake in male-related activities

Failure as a Father

Loss of self-worth

Inability to carry out stereotypical male job roles

Father figure comparison

31 PAU: Yeah. Yeah. I was also worried about seeking help initially as I feel the health services are not male friendly. I feel that males are meant to be the ‘strong alpha male’ and so we are perhaps treated less sympathetically than females? There are so many support groups out there for CFS which are targeted at mothers and often classes like yoga which I feel is female dominated. My male friends would definitely laugh at me for doing yoga anyway [laughs]

39 INT: [laughs] I mean, I shouldn’t laugh as that is such a shame as I know how much yoga can help to ease symptoms.

42 PAU: Yeah. My I do do it actually wife my wife, but of course don’t tell my male friends [laughs]

44 INT: Is your wife supportive?

45 PAU: Yes, she is. Although, I hope I can say this on record [laughs] but my um relationship with her has hindered slightly since I was diagnosed [INT: oh right-] Yeah. Yeah, well we have been trying for a second child for a while now, but the medication I take for my anxiety causes side effects that lowers my libido, on top of my fatigue. So, I always feel so bad that I don’t give her the intimacy she wants. I almost feel guilty, like I’ve stolen something from her by me being ill? I worry then that she will stop loving me. I feel like a failure as I can’t provide a big family that me and my wife would love. I do have a son, (NAME) but even that, I can’t go and watch him play rugby as standing up outside for a long time sets off my achy legs and painful joints. [INT: Yeah] So yeah, that really sucks because I know that all of his friends, all of their Dads go and watch their sons play rugby. My wife doesn’t understand rugby so she’d be useless! [laughs] Also, I can’t even like mow the lawn and that’s, well I feel anyway, a standard job that the male should do in the family. Maybe this is an old-fashioned way like in terms of when the men went to war etc, but males are seen as the main earner of the family and I haven’t worked now for years.

Failure as a husband

Inability to perform mundane tasks

Condition affects work performance/

ability

Lack of understanding from employer

Acceptance difficulties in terms of lifestyle changes

Lack of control over life plans

Feels males are the main earner

Hasn’t worked for years

Crippling fatigue and brain fog

Can’t help the wife out with food shopping

Suffers with memory difficulties

Used to work in a school as a teaching assistant full-time

Used to train in the gym every night

Had to move to part-time but that was a struggle

Boss was supportive

Suffered with glandular fever- possible cause as didn’t recover

Long-term fatigue

Used to be so active

Took a while to accept illness and that his old life was no longer

Planned to go into the army

Couldn’t pursue career aim

66 INT: Yeah. Yeah, I get what you mean.

67 PAU: I just can’t work due to my crippling fatigue and brain fog. That reminds me, like I can’t even help my wife out and do the food shopping as I forget like what brands to buy and things we need.

71 INT: Aw yeah, that must be tough. Where did you work before?

73 PAU: Well, I used to work in a school as a teaching assistant. I was working full-term, as well as training at the gym every night. But then when I gradually started to become ill and notice the CFS symptoms I went to part-time, but then even that was a um struggle. I mean I literally told my boss that I either go part-time or nothing at all like as I knew he wouldn’t understand why I need time to rest [laughs]

80 INT: Yeah ok. Do you think there was any particular reason for why you developed the condition?

82 PAU: Um, well I did suffer with glandular fever when I was in my early 20s, so I feel maybe that was it? I just didn’t recover and the fatigue just stayed with me. But I always used to be so active, so it took a while for me to accept that my old life was no longer. I was planning on going in to the army, so I really struggled to come to terms with the fact I couldn’t pursue my career aim.

89 INT: Yeah, I bet. [PAU: Yeah] Ok, well is there anything else you would like to add about your overall experiences?

91 PAU: Nope, that’s it for me.

90 INT: OK, well thank you so so much. I really appreciate it-

**Participant 3- SAM**

1 INT: Okay, so that's recording, isn't it?

2 SAM: Yeah, yeah. Perfect.

**Initial phrases**

**Emerging themes**

****3 INT: Okay. So first of all, start back to how you sort of felt prior to receiving a diagnosis. Just talk about, you know, initial symptoms, what made you seek a diagnosis? That sort of thing. How long ago was it?

Student studying photography

Tried to battle through the symptoms

Drinks one litre of energy drinks to stay alert

Muscles his way through- unsure of what symptoms were

Symptoms didn’t go away

Had depression/stress as a teenager

Doctors assumed depression was cause

Prescribed anti-depressants

Saw a psychologist

Continued to try to figure out symptoms

Relief and optimistic

Hope

Relief

Optimistic

7 SAM: It was about a 11 years ago that I started experiencing extreme fatigue. And all of a sudden, I remember, I was driving to studying photography at the time, and I was driving to class and I just had to pull over to the side of the road, and I realised I just couldn't even get to class. And um, I was kind of just trying to battle through it. [INT: Yeah] I was drinking like one litre of energy drinks before every class to try and like, stay alert. I was just like, I just tried to muscle my way through my hectic lifestyle. And then just, it just didn't go away. Um. And so I've kind of been involved in the mental health system with issues like depression and stress as a teenager. [INT: Hmm mm] And so my doctors, of course, just assumed it was depression and gave me antidepressants. And I even saw a psychologist for a little bit through the public system. And they, [laughs] but let's say, I was trying to figure out what was going on. And my partner at the time was trying to figure it out as well, and I think it was her that came across chronic fatigue first. And when I remember kind of looking through the list of kind of symptoms, and what was involved, and I went, yeah, that's actually finally a sufficient, that actually explains kind of my experience. [INT: Yeah]. Yeah. relieved. And I was initially kind of optimistic because you really, we …. and they, you know, have various medical practitioners’ kind of have like a uhhh kind of um overall package that they suggest. Yeah. And I was quite optimistic that by changing some things, um my symptoms will go away. It's hard to know what counts in the formal diagnosis, because my GP, I think it's probably similar system to the UK, my GP, kind of accepted and treated me as if I had chronic fatigue, but I still think he probably even 10 years later, probably thinks that is probably an element of psychological stuff going on. [INT: Yeah]. So, so from him, I don't think I would necessarily even today, get a proper formal diagnosis. Yeah, I've got another doctor I see about meds and stuff, and they wrote me, oh I actually, I must have got a proper diagnosis from someone about six or seven years ago. But it's one of those things where it's getting an official diagnosis, and who's required to make that diagnosis. Or, like I literally 10 years later, and I'm in I'm in a in a registered social worker. So, you'd think I'd know these kind of things. I don't know. [laughs]

Avoidance behaviour

Relief and optimistic to have a diagnosis

Sense that health professionals do not accept the illness

Lack of competence surrounding the diagnostic system

Hesitancy regarding the level of previous medical support gained

Optimistic that treatment will deter symptoms

Thought GP though CFS is an element of psychological stuff

Diagnosed properly 6/7 years ago

Wary over who has the power to make that formal diagnosis-

Had various tests to rule out other diagnoses- Diagnosis of exclusion

Had every test under sun

Had blood tests

A sense that should have pushed for more specialists

48 INT: Yeah, that is frustrating. Did you have any other other tests? Because I know um here and like myself, I think it's sort of you've got to sort of have symptoms for at least six months to a year, and then other tests are done to rule out any other diseases etc. So all the blood tests and everything.

51 SAM: Yeah. Yeah. So, my GP and I have kind of gone through and tried to test for everything we possibly can. And it's yeah, it's a diagnosis of exclusion. So, yeah, I've had every test under the sun. I haven’t really seen specialists and perhaps I should have pushed more for other specialists. But I've had all the bloods and tests for other kinds of infectious diseases and all sorts of stuff and yet nothing has ever kind of come back to say that something is wrong or abnormal.

58 INT: Yeah. Do you think that by not pushing to to get a referral to a specialist, do you think it is because you, you maybe feel that they don't believe you?

61 SAM: Um. It was more so the lack of energy I had. Waiting for referrals and travel to clinics is just so time-consuming. Yeah. And I've been kind of I've been studying and doing other stuff. I've got a kid as well. So yeah, I kind of just manage my condition the best I can [INT:yeah]. But perhaps getting a specialist hasn't been perhaps as much of a priority as as it should have been.

Continues to study- avoidance behaviour

Further reflection on past actions regarding gaining professional support

Appreciation towards other CFS cases

Awareness of symptom severity level

Fatigue prevented seeing medical advice

Busy studying

Got a child

Manages condition the best he can

Feels should have seen more specialists

Condition is not highly severe- feels lucky for that

Energy envelope/window

Crashed many times and been bedbound

Thankful for not having fibromyalgia

Fatigue is the main symptom

Part-time PhD Student

68 INT: Yeah. Do you think that your condition has sort of got worse over time? Or does it come in, you know, waves of severity?

71 SAM: Yeah, so I definitely have never been as severe as kind of some people are, so I am quite lucky there. So, I've never been I've been bound for, like weeks.

74 INT: Yeah.

75 SAM: But it's um definitely related to how hard I push myself and staying within that energy kind of envelope/ window. So yeah, there have been many times where I've crashed and been bedbound for like a week or two. Yeah, so it can get quite bad. I don’t have the Fibromyalgia joint and other kind or pains, which I am SO thankful for. It’s mainly the fatigue for me.

82 INT: Ok, so I remember you saying that you're a part time PhD student? Is that because that's probably still more manageable doing part time, than full time-

85 SAM: Yeah. Yeah. So, I pushed myself to do full time study through my bachelor's. And I kind of just muscled my way through it. And I definitely harmed my health massively. Like I finished my degree six years ago, and I still think my health is still getting over it. [INT:Yeah]. But we had to do things like a full-time social workplace, which was 36 hours a week. And then we're expected to do coursework on top of it. And there were some minimal accommodations from the university, for me having chronic fatigue, but it just wasn't really taken seriously. [INT: Yeah]. And I didn't want to kind of put my life on hold for years and years and years to do it part time. So, I just decided to try and get through it. And as a result, I'm kind of I think I'm still paying the price of that.

Studying full-time harmed his health massively

Minimal accommodations from the university

Felt he wasn’t taken seriously by University staff

Tried to get through it but still paying the price

Always has to weigh up the pros and cons

The condition dictates his life

Supportive partner but not mother and siblings

Don’t get the day-to-day reality of it

Mum tried to offer complementary therapy

Aware that avoidance behaviour has caused health consequences

Lack of support from the university

Life revolves around the condition

Lack of understanding by family

Lack of trust in effectiveness of complementary therapies

98 INT: Yeah, it's tough, isn't it. You've got to sort of weigh up the pros, the positives and negatives of doing something.

100 SAM: Yup. Always weighing up the pros and cons of what I should do. It dictates my life.

102 INT: So, how do you, have you got support from family and friends? Do they sort of understand it? I know from myself, my mum suffered with it when she was younger. So that is quite nice to know this, you know, they believe you. But do you feel that you have support around you?

107 SAM: My partner is really supportive, really good. Completely kind of gets my energy level. We've been together for quite a long time, so she's terrific with it. Yeah. My parents, my mom and my kind of siblings, I don't think they get it. They simply don't get the kind of day-to-day reality of it. And just yesterday, my mom was trying to promote some kind of energy healing type crap [INT: [laughs]] and was like “I’ve got a friend who had something similar to you and they were cured using this” and I was like “yeah right” [laughs] So um, I think, like my doctor, I still think they probably think that it’s psychological.

118 INT: Yeah, I think you know, my friends that they, they are aware of it and they try to understand but I think you just can’t understand until you've experienced it yourself. And I have been thinking maybe with the coronavirus pandemic, that might lead to some insight, you know, long COVID and everything.

Lack of understanding

Hopeful in terms of long Covid-19 related research

Lack of appreciation by employment staff

Unconscious assumptions of male competence

Speculation over gender differences in terms of how CFS is perceived

Inability to comply with the New Zealand culture

Hope that people will start to believe the condition is real

He’s a staunch feminist

Believes the oppression of women systemically across society

Noone takes it seriously at the University

Assumption of competence in being a white male

Men can just do stuff and that they have to be strong

Discounted because a male

New Zealand culture causes men to be reluctant to share emotions

124 SAM: Yeah, so hopefully, people might start to believe the condition. Yeah and, um, I was thinking in terms of your research project, I’ve been kind of reflecting on masculinity and chronic fatigue. So, I'm a staunch feminist and I strongly believe the oppression of women systemically across society. So I'm not like trying to make any kind of say that women have it better because they certainly don't, but I think in terms of the chronic illness I have, I was thinking about it because I'm employed part time at the university and literally no one takes it seriously. I've said “look, I've got chronic fatigue, I see doctors and specialists about it, here's how it impacts my life etc”. And I've been wondering whether being a white male, there's that assumption of competence. And um whether if I were a female, they might go “ah, chronic illness, that is something which is real”. But they look at me and see a man and have those kind of unconscious assumptions that men can just do stuff and that they have to be strong. [INT:Yeah] Yeah, and so I'm wondering if the patriarchy is kind of leading to my illness being discounted in a way which would be different if I were a woman? Yeah, that's just pure speculation. But what I can say about masculinity and in New Zealand there is that blokey culture and men are very kind of reluctant to share when they're struggling with their emotions and so I felt I could not share anything.

150 INT: Yeah, that's what did prompt me to do this research. You know, it is known that males are less likely to seek help, especially for mental health. So, is that a reflection on a condition like this, that is perceived to be psychological?

Stereotype that men do not open up about emotions as this means a loss of masculinity

Inability to succeed to best standard

More open with it with my female friends

Believes there is a tough male culture in New Zealand

Highest male suicide rates in New Zealand

Blokey culture

Feels that supervisors accept that he can’t produce as much work as he would like, but don’t understand the condition

Use CFS when referring to the condition

154 SAM: Thinking about it, I've been much more open with it with my female friends, then my male friends, which probably does reflect the idea of the tough male culture, which is really prevalent in New Zealand, and we've got, New Zealand's got one of the highest male suicide and highest youth suicide rates in the world. We're very blokey culture. And so I think it's just not really done that men, open up. And so I think that that has been reflected in my experience that I haven't opened up to other guys about it.

162 INT: Yeah. Yeah. It is interesting.

163 SAM: Yeah, like, I feel like my supervisors, accept that I can't produce as much work as I would like, but they just don't understand what our chronic fatigue is or what it's like to kind of experience it.

167 INT: Yeah. And there's a lot of people that think, you know, well, just sleep more or maybe it's also that chronic fatigue it sort of just sounds like, you're just not sleeping, you know, you're you're staying up late. Yeah, it's funny, do you, if people ask you do you do you refer to it as chronic fatigue? or M.E? Chronic fatigue, it's obvious what it is then but M.E people aren't really, they're not sure. Are they-

173 SAM: I always use chronic fatigue just because it's a more accurate reflection of my experience. And I don't know if I have M.E because that refers to the swelling in the brain, right?

177 INT: Yeah. Maybe by the condition having 2 names that it is referred too accounts for the stigma because people are like “well which one is it”? etc.

180 SAM: Yeah definitely. So in terms of, like masculinity that I was reflecting on being a male is like unconsciously I am aware that this is like a stereotype and one that needs to be challenged. I felt I needed to be that male breadwinner and want to kind of provide, want to be a successful Father for my kidl. I'm sure it's the same for woman too, but I'm sure there are variations too, for in terms of being a male and experiencing that. And so there is that kind of feeling of like, because I've got a kid and I've got this, and things, I've kind of like letting the family down to some extent because I can’t be the primary earner and I can’t do some of the things that would kind of be expected. And like New Zealand kind of culture, people do a lot of activities outdoors I'm very limited in the degree to which I can do these things. The kind of things that guys would do around me like we go outdoor stuff or play sports, that sort of thing, it’s simply impossible with chronic fatigue. I wasn't sporty before my diagnosis, I was I was into other stuff, like camping and rock climbing. But I, I couldn't any longer go on protests or be involved in the animal rights activism I was doing.

Stereotype of masculinity

Sense of failure as a Father

Limited in what physical activities can do

Loss of previous hobbies

Loss of hobbies

Acceptance of symptom control

Aware that there is a stereotype of masculinity

Male breadwinner has to be successful

Feels he his letting the family down

Can’t be the primary earner

New Zealand- outdoor activities are common

Impossible to do sports

Used to camp, rock climb, animal campaigning

Watches a lot of TV and doing bonsai trees as a coping mechanism

200 INT: Yeah. Yeah. Which is a shame because well, you know, you still need to do your sort of hobbies and things that you enjoy for your mental health. So, it's keeping that sort of balance. [SAM: Yeah] So what are your preferred coping mechanisms to the fatigue?

155 SAM: Um, I do watch a lot of watching TV series. That's pretty much my go to for when my brain just has to rest. I've got a background of horticulture and I used to do community gardening and gardens and things like that. And I can't do that anymore because of the fatigue. So, I've now got into bonsai trees, so I can work on them at my own pace sat down in the garden [laughs]. And it's been really interesting, because I've gotten to it in the past year, and that is almost exclusively guys. And it's a really positive male community actually, like everyone is super chilled and supportive, it feels quite different. So that's been a really quite positive hobby to pick up and be involved in.

Involved in a supportive, male based activity

Pandemic and working from home has increased fatigue level

Intersection of masculinity and chronic illness- not considered before

Reflects on whether treatments are given differently in relation to gender

Condition viewed as socially disabling in females

Lack of recognition of non-physical suffering

Feels the invisible nature means illness is not noticed or recognised

Only females spoken to him about mental health

Found a supportive male community

Gender differences in illness perception and treatment

Male competence causes illness to not be as socially disabling

Lack of recognition for the illness

INT: Oh that is good. Yeah.

158 SAM: In terms of the pandemic, working from home has enhanced my fatigue as having my son and partner around causes more noise and stimuli etc. So, it is hard for me to rest.

160 INT: Yeah, I can understand that! Thank you for all of the points you have been making. Is there anything else you would like to add?

162 SAM: Yeah, it's been really interesting for me, thinking about the intersection of masculinity and chronic illness is something I hadn't really considered before. And, yeah, just kind of reflecting on whether I would have been seen or treated differently if I were a woman and what being a man has led to. And I think in some ways, I haven't been held back as much and that my boss kind of sees me and ignores the fact that I've got a chronic illness. And so, I think the whole kind of assumption of competence as a male has kind of, to some extent, worked in my favour in terms of um my chronic illness not being as socially disabling as it might have been if I were a female. But then in other ways, I think it has kind of been difficult for me being a male due to the lack of recognition of non-physical suffering. I think the invisible nature of the illness means it is not noticed or recognised. Also, I've had several female friends have opened up to me about their mental health struggles. I don't know if a single man in my life is ever opened up about the fact that they're mentally struggling and I think this would be the same with chronic fatigue. I have certainly have only told females about my condition and no males. [INT: Yeah]. I mean males have told me that life is tough for them, but they never go into details about their suffering etc.

Inability to do desired physical activities

The need to constantly forward plan as a symptom management strategy

Stereotype for males to hide emotions

Not taken seriously by others

Men are reluctant to seek medical advice

Male competence causes reluctance to seek help

Only told males about his condition

Partner understands

Can’t do physical things

Has to be careful about what activities he does

Has to work out what to say to his son

Believes that autoimmune disorders are more common in women

Hormones

Females are easily emotional and are encouraged more to talk about their emotions

Contraception

Feels that males are not taken seriously

Believes that CFS is female dominated

‘man-up’- causes reluctance

Afraid to go to the doctors as worry what friends would think

177 INT: Yeah.

178 SAM: My partner understands that I can’t do physical things but like my son, he wants to go mountain biking, and all that kind of stuff. Stuff which I have to be very careful about what I do. I don’t want to say I’ll do something with him and then when it gets to the time I can’t do it because I’m too tired. I feel I always have got to work out what you’re going to say.

182 INT: So, just one more question ok. What are your thoughts on why CFS is more prevalent in females than males?

184 SAM: Well, I think autoimmune disorders are more common in women, so maybe it is due to biological factors? Perhaps it’s due to hormones as well. I know that females do get easily emotional when they go onto hormone contraception, so perhaps they find it easier to talk about their emotions or people can physically see they are in distress and so are encouraged to talk about their symptoms more so than males because they are more inclined to hide their symptoms? There has been so much publicity on how makes are not taken seriously when they admit how they are feeling, so this puts me off going. I actually have a male friend now who suffers with depression, but whenever I ask how he is, he shrugs it off and says he is fine. I also think that CFS is ‘female dominated’ and that men are often less likely to seek medical help for any condition unless it is life-threatening. [INT:Yeah] Also, the phrase of “man up” often make men reluctant to seek a diagnosis. I was afraid to go to the doctors about my symptoms as I was worried that my male friends would take fun out of me and tell me to stop moaning… but yeah, my family aren’t that supportive really. They just simply don't get the kind of day-to-day reality of it and I think because I don’t know any other males with it, I can’t get them to like tell my friends and family that they go through the same thing.

108 INT: Thank you they are good points, well that’s everything I have to say. We’ve covered a lot.

110 SAM: Sure. You're welcome to flick me through an email if you have any other questions or if you want to set up a follow up interview, I'm open to that. I don't know what your ethics allows you to do. But just let me know if you do want to, if you've got anything comes to mind, particularly later on in the project, you might go hang on a second, I wish I'd asked that. So, feel free to get in touch.

117 INT: Oh, thank you [NAME]. I really, really do appreciate it. I am struggling a little bit to get participants so thank you. Right I’ll stop the recording now-

**Participant 4 Tyler**

**Emerging Themes**

**Initial phrase**

****1 INT: Ok, so that is recording now. Before we start, have you any questions?

3 TYL: Nope, all good with me. I can hear you and see you so that is good [laughs]

Had a car crash in 1994

Diagnosed with epilepsy- possible cause?

Thought medication was causing the fatigue

GP ran tests- ECGs

Everything came back normal

Complained of lower back pain

Diagnosed with arthritis and spondylitis

Didn’t agree with diagnoses

Spoke to a specialist

Suffers with insomnia and muscle aches/pain

Tests demonstrated body inflammation

Unaware of what M.E is

Various diagnoses

Recognition of CFS from HP’s

4 INT: Yes, perfect [laughs] Okay, so um, so yeah, so just tell me about your experiences prior to getting the diagnosis. Did you know that it probably it was chronic fatigue?

7 TYL: Well I had a car crash in 1994 and I was diagnosed with epilepsy, maybe as a result of that I’m not sure. [INT: Okay]. And I was taking medication for that at the time and I thought that that was making me tired all the time, even though it stopped the seizures. So, I checked with the GP and they ran lots of tests like ECGs, but everything came back normal. But I just knew that it wasn’t side effects of the tablets, it was something else. So after still complaining of lower back pain and fatigue, I got diagnosed with arthritis. I also then [laughs] got diagnosed with spondylitis [INT: Ah, ok], but I just didn’t think that I came into that category so I had another chat with the specialist and they said that the things I’m describing, like I can’t sleep and the muscle aches and pain, he thought that sounded like M.E. So he ran some tests and found I had high …… in my blood which means that some part of my body is inflamed. Mine was something like 28 and I think normal is like 6. [INT: Yeah gosh]. So, the doctor said, this sounds very much like M.E and I said “M.E, what’s that”? [laughs] He said it’s also called CFS, but there’s no medication for it apart from the generic painkiller.

27 INT: Yeah, so did you sort of get the impression from the doctor that he thought it was more of a psychological thing?

29 TYL: Yeah definitely. He called it ‘pseudo M.E’ and I was like no, this is not in my mind. I am not making it up or imagining the pain. Before going to the doctors, I remember just trying to battle through it

Sense that CFS is not real

Lack of understanding of the condition by HP’s

Distrust towards some HP’s, but not all

Lack of support for males

Inability to carry out mundane tasks

Felt that doctors see CFS as a psychological illness and that one is making it up

Took eight years to get a diagnosis

Doctors thought symptoms were due to a lack of water intake

Treated properly in Cornwall, compared to Southampton

Provided with information on how to deal with CFS.

Relief

Feel CFS is more common in females

Lack of forums with males

Can’t do hobbies in which used to do

Have to go to bed straight after doing minimal activity

Likes to crochet

Brain seems to be on all the time

Suffers with sleeping problems

33 INT: Yeah, that's interesting. I've never heard of pseudo M.E.

34 TYL: It took me eight years to get diagnosed with M.E. I remember once the doctor said my symptoms are because I am not drinking enough water and I said “No, I drink 2 litres of water a day” [laughs]. [INT: Yeah]. I came down to Cornwall then however and it was a completely different scenario and I finally found like I was treated properly and listened to compared to when in Southampton. So I saw a specialist and he kind of gave me a set information on how to deal with um CFS.

43 INT: Ok yeah, that is good-

44 TYL: So, I then FINALLY got a proper diagnosis of CFS stroke M.E. Yeah, apparently, it's very rare for men to have it and females are more likely to have the condition due to their genetics. But I feel there's not very many forums for men to talk about M.E?

49 INT: Ok, let’s go back to that again later if you don’t mind as that is a good point. [TYL: Sure] So, it seems as if it has impacted on on your life significantly and, and, you know, you can't do the hobbies you would have would have liked to have done or maybe used to do.

54 TYL: Yeah, well I mean even when I go to cut the lawn, after that I would have to go straight to bed and rest. I’ve also been crocheting a lot recently, mainly because it rests my brain and calms me. I find I sleep better after doing it as well. But my brain seems to be on all the time, it just doesn’t switch off [laughs]. But yeah, I feel that there aren’t many mediums out there for men to discuss problems like sleeping etc.

Feels there aren’t many mediums out there for male support- groups are always targeted for women

Doctor said he was unique to be a male with the condition- reluctant to find another male to talk too

Grew up surrounded by women

Didn’t have a male model

Female led household made him stronger- Experienced women opening up about problems

Husband understands and accepts his condition

Friends and other family members don’t understand

Worries about telling friends and family about condition

Condition affects social life- worries that others think condition is an excuse

Deals with others

Speculation that there is a low CFS prevalence in males

Lack of male role model

Talking about emotions/physical illness relates to past experiences

Support from significant other

Lack of support from friends and family members

Negative impact on social life

63 INT: Ok, so why do you think that?

64 TYL: I think it's mainly because of support groups I feel are always targeted for women? I mean I can’t be the only male in the world who has CFS. Even my doctor told me that I am quite unique in the sense that I am male with this condition. So I think then, that just instantly made me feel that it will hard to find another male that is willing to discuss their experiences. You know?

71 INT: Yeah. So do you think that maybe males they don't speak out as much because they are perhaps perceived to be strong and and not be weak?

74 TYL: Yeah, I think so. I grew up surrounded by all women and so I didn’t really have a male role model as my Dad was out at sea a lot. But on the other hand, because I grew up in a female led household, I think this made me strong and I was always included when they would open up about their problems. So yeah, I am now aware of the importance of speaking out about your emotions etc. Luckily, my husband is very understanding with me and accepts my condition.

82 INT: Does the rest of your family and friends understand-

83 TYL: No, they don’t really. I get worried about telling them as I know what their reactions will be. For example, ‘Oh we all get tired in the afternoon’ or ‘oh it’s your age’. But having my diagnosis made me stronger in my mind. [INT: Yeah]. I know that my condition affects my social life as talking to other people just takes my energy away. I is also tiring just worrying about whether they think I am just using my condition as an excuse to not see them. [INT: Yeah]. So you are not only dealing with yourself, you are dealing with other people questioning you.

93 INT: Yes, I totally understand what you mean by that. I think it makes it harder when the condition just isn’t well known by the public. Also, you can’t see it [laughs] it’s not like a broken leg.

Feels can open up and share experiences with others with the condition

Strong minded

Talking about the condition helps

Feels there should be support groups just for males

Every aspect of life is affected

Gets pins and needles

Aware of more symptoms when know what others suffer with

True friends are the ones that have stuck by him

Lost more male friends than females

Feels invisible

Old life has been snatched like a punishment

Determination to continue with life as well as can

Variation of symptoms deters daily activity

Loss of belonging

97 TYL: Yes, I know. Even talking to you know with knowing that you suffer with the condition, I can really open up and share my experiences that I wouldn’t necessarily share with others as I know they just won’t understand! [INT: Yeah]. ‘I won’t let my M.E rule me’ is my motto that I tell myself.

102 INT: Oh, I love that. That is very true. A very good quote [TYL: Yeah, I think so].

104 TYL: Talking about the condition definitely makes me feel better and so I do think there should be some support groups made solely for males. Every aspect of my life affected, even driving, going to the shops etc. I get pins and needles a lot recently, symptoms seem to be ever changing and talking to people about the variety of symptoms is useful. Sometimes you don’t realise symptoms that you have, but when others mention it like online forums you think “oh yeah, I do have that actually, that explains it”.

113 INT: Yes, 100%. Ok well thank you so much for all of that. Have you got anything else you would like to add?

115 TYL: So with regards to the concept of male experiences, I feel that since my diagnosis my male friends that have stuck by me, they are my real friends, but I have definitely lost more male than female friends. Hmmm. But like yeah, sometimes I feel invisible, and my old life has been snatched from me like some kind of punishment.

121 INT: Aw, yeah. Yeah. I am sorry for you’re suffering. It is an awful illness. Well, I will just stop the recoding now so my recordings don’t get too big of a file size-
